# Inhibition of Monoamine Oxidases by Pyridazinobenzylpiperidine Derivatives

**DOI:** 10.3390/molecules29133097

**Published:** 2024-06-28

**Authors:** Jong Min Oh, Yaren Nur Zenni, Zeynep Özdemir, Sunil Kumar, Semanur Kılıç, Mevlüt Akdağ, Azime Berna Özçelik, Hoon Kim, Bijo Mathew

**Affiliations:** 1 Department of Pharmacy, and Research Institute of Life Pharmaceutical Sciences, Sunchon National University, Suncheon 57922, Republic of Korea; ddazzo005@naver.com; 2 Department of Pharmaceutical Chemistry, Faculty of Pharmacy, Inonu University, Malatya 44210, Türkiye; yarennur.zenni@inonu.edu.tr (Y.N.Z.); semanurk489@gmail.com (S.K.); 3 Department of Pharmaceutical Chemistry, Amrita School of Pharmacy, Amrita Vishwa Vidyapeetham, AIMS Health Sciences Campus, Kochi 682041, India; solankimedchem@gmail.com; 4 Department of Pharmaceutical Chemistry, Faculty of Pharmacy, Afyonkarahisar Health Sciences University, Afyonkarahisar 03030, Türkiye; mevlut.akdag@afsu.edu.tr; 5 Department of Pharmaceutical Chemistry, Faculty of Pharmacy, Gazi University, Ankara 06100, Türkiye; azime@gazi.edu.tr

**Keywords:** monoamine oxidase inhibitors, pyridazinone, benzylpiperidine, molecular docking

## Abstract

Monoamine oxidase inhibitors (MAOIs) have been crucial in the search for anti-neurodegenerative medications and continued to be a vital source of molecular and mechanistic diversity. Therefore, the search for selective MAOIs is one of the main areas of current drug development. To increase the effectiveness and safety of treating Parkinson’s disease, new scaffolds for reversible MAO-B inhibitors are being developed. A total of 24 pyridazinobenzylpiperidine derivatives were synthesized and evaluated for MAO. Most of the compounds showed a higher inhibition of MAO-B than of MAO-A. Compound **S5** most potently inhibited MAO-B with an IC_50_ value of 0.203 μM, followed by **S16** (IC_50_ = 0.979 μM). In contrast, all compounds showed weak MAO-A inhibition. Among them, **S15** most potently inhibited MAO-A with an IC_50_ value of 3.691 μM, followed by **S5** (IC_50_ = 3.857 μM). Compound **S5** had the highest selectivity index (SI) value of 19.04 for MAO-B compared with MAO-A. Compound **S5** (3-Cl) showed greater MAO-B inhibition than the other derivatives with substituents of -Cl > -OCH_3_ > -F > -CN > -CH_3_ > -Br at the 3-position. However, the 2- and 4-position showed low MAO-B inhibition, except **S16** (2-CN). In addition, compounds containing two or more substituents exhibited low MAO-B inhibition. In the kinetic study, the K_i_ values of **S5** and **S16** for MAO-B were 0.155 ± 0.050 and 0.721 ± 0.074 μM, respectively, with competitive reversible-type inhibition. Additionally, in the PAMPA, both lead compounds demonstrated blood–brain barrier penetration. Furthermore, stability was demonstrated by the 2V5Z-**S5** complex by pi–pi stacking with Tyr398 and Tyr326. These results suggest that **S5** and **S16** are potent, reversible, selective MAO-B inhibitors that can be used as potential agents for the treatment of neurological disorders.

## 1. Introduction

**Figure 1:**
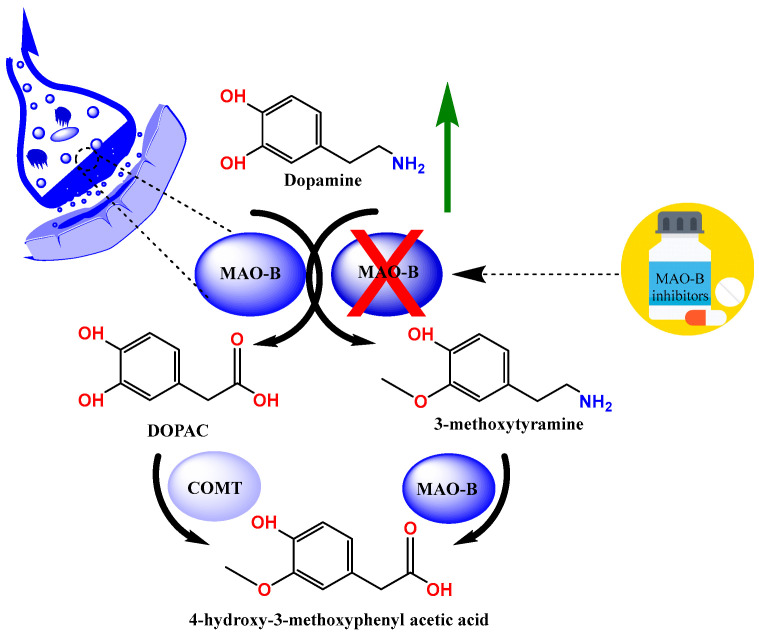
The metabolism of dopamine.

Parkinson’s disease (PD) is a neurodegenerative disease that is particularly common among the elderly and is characterized by the death of dopaminergic neurons in the substantia nigra pars compacta [1,2]. The typical motor features of PD (bradykinesia, stiffness, and tremor) are caused by a subsequent dopamine shortage; however, non-dopamine neurodegeneration results in additional non-motor symptoms that manifest at different times [3]. The pathogenesis of the disease is characterized by elevated acetylcholine and low dopamine levels; therefore, treatment strategies aimed at increasing dopamine levels are recommended. One such strategy is the inhibition of monoamine oxidases (MAOs), which are responsible for the metabolism of endogenous amines (Figure 1) [4,5,6,7].

MAO inhibitors (MAOIs) have been crucial in the search for anti-neurodegenerative medications and continue to be a vital source of molecular and mechanistic diversity [7]. Therefore, the search for selective MAOIs is one of the main areas of current drug development. To increase the effectiveness and safety of treating PD, new scaffolds for reversible human MAO-B (MAO-B) inhibitors are being developed [8,9,10,11].

**Figure 2:**
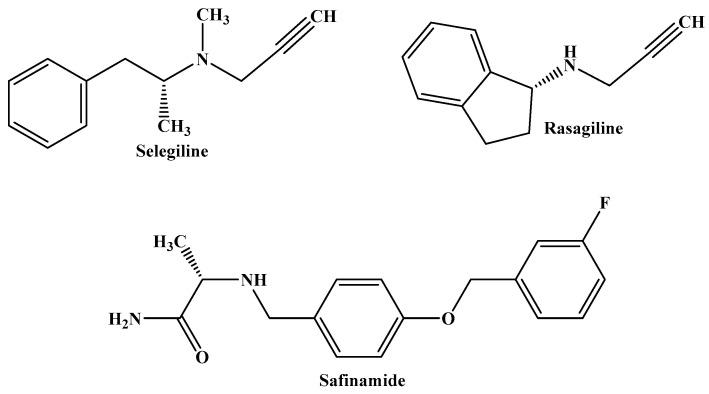
The molecular structures of selegiline, rasagiline, and safinamide, which are MAO-B inhibitors currently used in the treatment of PD.

Selegiline and rasagiline are MAO-B inhibitors used to treat levodopa-induced motor fluctuations, advanced PD, and the loss of OFF time in patients. Safinamide, a third MAO-B inhibitor, is used as an adjuvant therapy or as a dopamine agonist in conjunction with levodopa, and it combines additional non-dopaminergic qualities that may be beneficial for patients with PD (Figure 2) [12,13,14].

**Figure 3:**
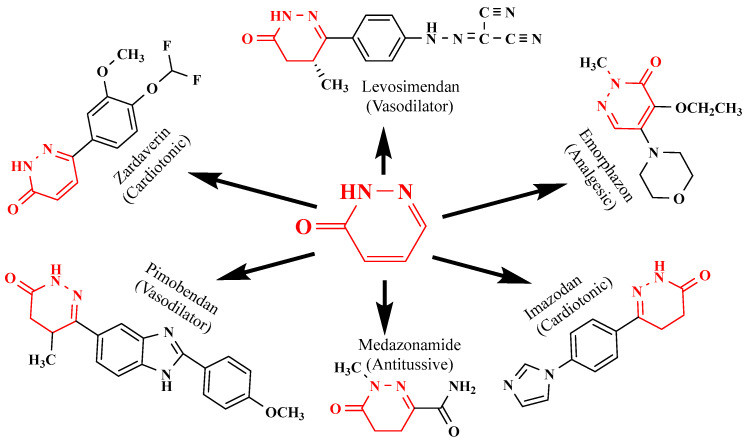
Structure of pyridazinone derivatives.

The pharmacological properties of pyridazinone analogs include anti-inflammatory, anticancer, antibacterial, anticonvulsant, analgesic, antioxidant, antihypertensive, antisecretory, and antiulcer effects [13,14,15,16,17,18,19,20,21]. They include lipoxygenase inhibitors, cholinesterase inhibitors, adenosine receptor antagonists, serotonin receptor antagonists, phosphodiesterase inhibitors, glutamate transporter activators, dipeptidyl peptidase inhibitors, cyclooxygenase (COX) inhibitors, and vasodilators [22,23,24,25,26,27,28,29,30]. Additionally, a few commercially available drugs, including pimobendan, levosimendan (a cardiotonic medication), emorphozan, and zardaverine (a non-steroidal anti-inflammatory drug) as analgesics and anti-inflammatory agents, are based on pyridazine rings (Figure 3).

**Figure 4:**
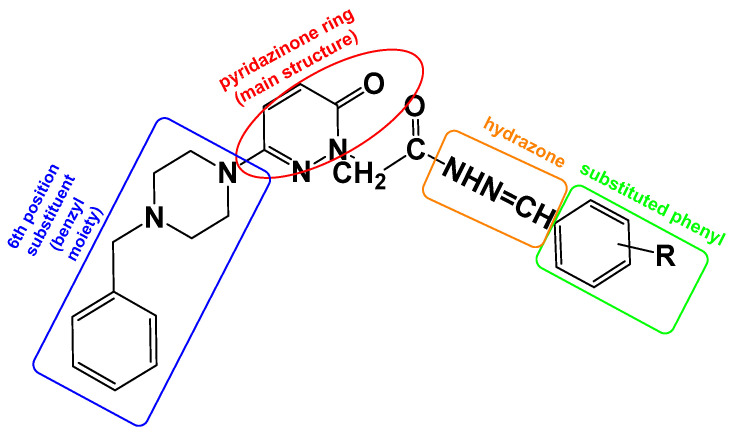
The design targets to be applied on the molecule within the scope of this study. The pyridazinone ring is protected as the main structure (red), containing the benzyl moiety at the 6th position of pyridazinone (blue), and hydrazone part (orange) and substituted phenyl moiety (green).

To increase the biological activity of pyridazinone, new pharmacological molecules known as benzylpiperidinopyridazine derivatives were synthesized. The resulting compounds were selective MAO-B inhibitors, and the activity results were corroborated by the molecular modeling scores (Figure 4).

## 2. Results

### 2.1. Chemistry

This study synthesized and investigated the in vitro enzyme inhibitor activities of 23 compounds that act as selective MAO-B inhibitors, which were obtained from 6-substituted-3(2H)-pyridazinone-2-acetyl-2-(substituted benzalhydrazone) [10,11]. Besides unsubstituted benzaldehyde, benzaldehyde derivatives with disubstituted methylamine, methyl, bromo, fluoro, methyl, and methoxy substituents at various positions were used in synthesizing benzhydrazone derivatives, and the corresponding contributions of these groups to the activity were investigated. The structures of the obtained compounds were confirmed using spectrum data from 1H-NMR, 13C-NMR, and TOF-MS. Their melting temperatures were determined, and the findings are shown in the Supplementary File and Table 1.

### 2.2. Inhibition Studies

#### 2.2.1. The Inhibitory Activities of MAO-A and MAO-B by the Compounds

Twenty-four pyridazinobenzylpiperidine derivatives against MAO-A and MAO-B were evaluated. These pyridazinobenzylpiperidine derivatives were synthesized with two large structures: one residue was attached to the phenyl ring, and two or more residues were attached to the phenyl ring (Figure 4). Seventeen of the twenty-four compounds showed higher MAO-B inhibition than MAO-A except seven compounds (Table 2). Compound **S5** showed potent MAO-B inhibition with an IC_50_ value of 0.203 μM, followed by **S16** (IC_50_ = 0.979 μM). In contrast, compound **S15** showed the highest MAO-A inhibition with an IC_50_ value of 3.691 μM, followed by **S5** (IC_50_ = 3.857 μM) (Table 2). The selectivity index (SI) of MAO-B over MAO-A was the highest for compound **S5** (19.04) and the lowest for compound **S9** at 0.62 (Table 2).

#### 2.2.2. Reversibility Studies

**Figure 5:**
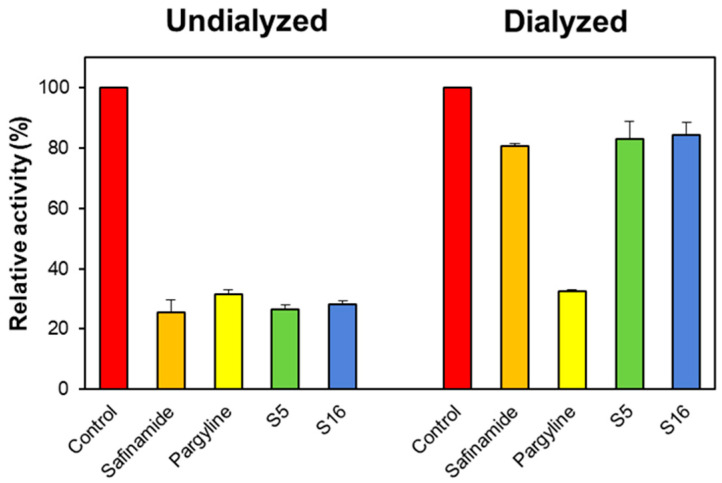
The recovery of MAO-B inhibition by **S5** and **S16** using dialysis experiments. The concentrations of **S5** and **S16** used were approximately 2.0 times their IC_50_ values (0.4 and 2.0 μM). After 30 min of pre-incubation, the mixtures were dialyzed for 6 h with two buffer changes.

In the reversibility study, the concentrations of **S5** and **S16** used were approximately 2.0 times their IC_50_ values (0.4 and 2.0 μM for MAO-B, respectively). The residual activities before and after recovery are represented by A_U_ and A_D_. Compounds **S5** and **S16** were recovered from 27.53% to 79.07% and from 29.04% to 81.33%, respectively (Figure 5). The recovery values of **S5** and **S16** were similar to safinamide (ranging from 28.34% to 81.19%), whereas they differed from pargyline (ranging from 32.66% to 32.93%). These results indicate that **S5** and **S16** are reversible MAO-B inhibitors.

#### 2.2.3. Enzyme and Inhibition Kinetics

**Figure 6:**
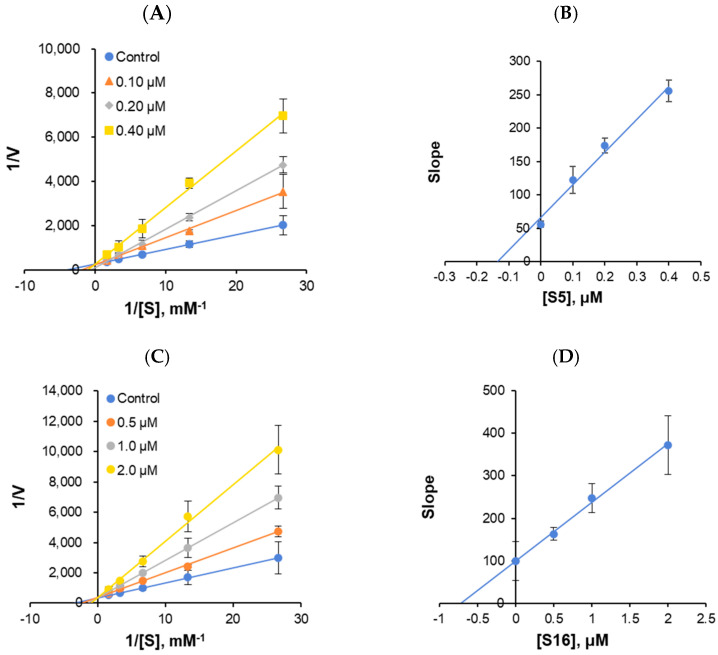
Lineweaver–Burk (LB) plots for MAO-B inhibition by **S5** and **S16** (**A**,**C**), and their secondary plots (**B**,**D**) of the slopes versus inhibitor concentrations. The experiments were analyzed using five substrate concentrations and three inhibitor concentrations.

The enzyme kinetics of MAO-B and the types of inhibition were analyzed at five concentrations of benzylamine as a substrate and three inhibitor concentrations. In the LB plots, **S5** and **S16** appeared to be competitive MAO-B inhibitors (Figure 6A,C). Secondary plots showed that the K_i_ value was 0.155 ± 0.050 and 0.721 ± 0.074 μM, respectively (Figure 6B,D), suggesting that **S5** and **S16** are competitive MAO-B inhibitors.

### 2.3. Parallel Artificial Membrane Permeability Assay (PAMPA) for Blood–Brain Barrier (BBB) Permeation Study

The results obtained from the PAMPA indicated significant levels of permeation and CNS bioavailability for compounds **S5** and **S16**. **S5** showed a Pe value surpassing 4.0 × 10^−6^ cm·s^−1^, while **S16** came close to this threshold, suggesting higher rates of BBB penetration at 5.75 × 10^−6^ cm·s^−1^ and 3.78 × 10^−6^ cm·s^−1^, respectively (Table 3).

The effective delivery of CNS medications relies on successful brain penetration. To evaluate the brain permeability of all the derivatives, PAMPA-BBB was used in this study. The extent of penetration was determined using a specific formula and considering the effective permeability of the substance (Log Pe). Compounds classified as potentially permeating (CNS+) exhibited a Pe value greater than 4.0 × 10^−6^ cm·s^−1^, while those categorized as likely non-BBB permeating (CNS−) had a Pe value less than 2.0 × 10^−6^ cm·s^−1^.

### 2.4. Molecular Docking

**Figure 7:**
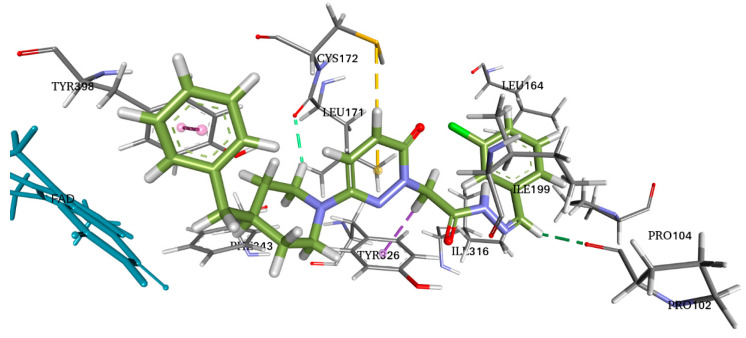

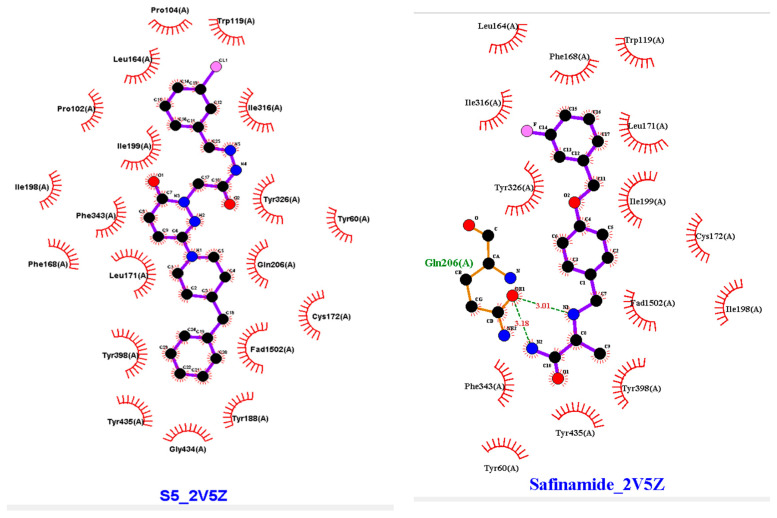
Two-dimensional (2D) and three-dimensional (3D) interactions of **S5** and safinamide with 2V5Z using LigPlot and Biovia Discovery Studio, respectively.

Molecular docking allows for the prediction of the molecular affinity at the binding site, important interactions, and experimental binding mechanisms. With this knowledge of the molecular interactions between the physical and chemical processes, new derivatives can be developed. Therefore, we studied the molecular docking of **S5** and 2V5Z (Figure 7).

## 3. Discussion

### 3.1. Chemistry

The initial step in the synthesis was a benzylpiperidine substitution at position six. The pathway begins with the commercially acquired 3,6-dichloropyridazine and proceeds in five steps to obtain the final products. Mass spectral data demonstrated that the substitution was performed unilaterally and that the first-stage product was obtained with a yield of 52%. The lactam carbonyl present in the pyridazinone ring at 1660 cm^−1^ in the IR spectral data was used to confirm the results of the hydrolysis of the pyridazine ring in an acidic environment, which was the following step. The SN2 reaction attacks the bromine-bonded carbon of ethyl bromoacetate, producing an acetate derivative. This attack was caused by the unshared electrons of the nitrogen atom in the second position of the pyridazinone ring. Given the ability of bromine to retain electrons, the second nitrogen of the pyridazinone ring attacks the electron-poor carbon atom in the basic environment produced by the presence of potassium carbonate. The ester derivative and hydrazine hydrate react to produce the acetohydrazide derivative, which was produced in the following steps. As the alcohol is stripped from the molecule, the hydrazine hydrate nucleophile hits the carbonyl carbon, initially causing an addition reaction, followed by an elimination reaction by the formation of a double bond between carbon and oxygen.

The final stage involved the reaction of acetohydrazide with both substituted and unsubstituted benzaldehyde derivatives to produce the final compounds. Similar to the previous synthesis step, the next step involves an elimination reaction in which water is released from the structure and a carbon–nitrogen double bond is formed. The addition reaction starts with the nucleophilic attack of the free electrons of the hydrazine nitrogen on the carbon of the benzaldehyde carbonyl. In derivatives from benzaldehyde derivatives with electron-withdrawing substituents on the ring, the resulting compounds were produced in yields as high as 97.37%.

### 3.2. The Inhibitory Activities of MAO-A and MAO-B by the Compounds

The IC_50_ value of **S5** for MAO-B was lower than a pyridazinone containing dithiocarbamyl moiety **12b_1_** (IC_50_ = 6.71 μM) [31], a 4-phenethyl-1-propargylpiperidine **15** (IC_50_ = 4.3 μM) [32], and a pyridazine–coumarin derivative **9b** (IC_50_ = 0.75 μM) [33] but higher than a pyridazinone derivative **TR16** (IC_50_ = 0.17 μM) [11], a pyridazinone containing the (2-fluorophenyl) piperazine moiety **T6** (IC_50_ = 0.039 μM) [10], and a pyridazinone derivative **4b** (IC_50_ = 0.022 μM) [16]. In addition, the IC_50_ value of **S15** for MAO-A was lower than pyridazinones containing dithiocarbamyl moiety (IC_50_ >100 μM) [31], pyridazine–coumarin derivatives (IC_50_ >100 μM) [33], a 4-phenethyl-1-propargylpiperidine **9** (IC_50_ = 15.5 μM) [32], and a pyridazinone derivative **TR8** (IC_50_ = 12.02 μM) [11] but higher than a pyridazinone containing the (2-fluorophenyl) piperazine moiety **T6** (IC_50_ = 1.57 μM) [10] and a pyridazinone derivative **4f** (IC_50_ = 1.05 μM) [16]. Regarding the structure–activity relationship (SAR), compound **S5** (3-Cl of phenyl ring) had 4.82–53.94 times higher MAO-B inhibition than other derivatives. When compared according to the position of the residue, MAO-B inhibition by 2-CN at the phenyl ring (**S16**) was higher than that by other derivatives with substituents of -CN (**S16**) > -Br (**S3**) > -OCH_3_ (**S19**) at the 2-position. In addition, the 3-Cl of the phenyl ring (**S5**) showed the highest MAO-B inhibition compared to the other derivatives, with substituents of -Cl (**S5**) > -OCH_3_ (**S11**) > -F (**S7**) > -CN (**S17**) > -CH_3_ (**S10**) > -Br (**S4**) at the 3-position. However, compounds with residues at the 4-position or two or more residues showed weak MAO-B inhibition (Figure 4, Table 2). These results suggested that compounds **S5** and **S16** are potent selective MAO-B inhibitors.

### 3.3. Molecular Docking

AutoDock Vina software was used for docking. Vina uses PDBQT molecular structure file types as both input and output. Vina created up to 30 configurations for each docking run, ranking them according to the largest energy difference (kcal/mol) between the optimal and lowest binding modes. The optimal docking scores for **S5** and safinamide in affinity were −10.4 and −11.0 kcal/mol, respectively. The **S5** and safinamide and **S5** bound to the same binding pocket with almost similar amino acid residues. In the case of a native ligand, the major interactions were hydrophobic and bipartite hydrogen bonds between the amide side chain and Gln206 residue. The binding study results indicated that a Pi–sulfur bond holds the pyridazine ring of **S5** to Cys172. Pro102 and Leu171 form a typical hydrogen bond with **S5**. Tyr398 and Tyr326 residues utilize pi–pi stacking to interact with the ligand (Figure 7). As noted above, **5S** suppressed MAO-B expression in vitro. Furthermore, we observed that hydrogen bonding and hydrophobic interactions stabilized the 2V5Z and **S5** complexes.

## 4. Materials and Methods

### 4.1. Chemistry

**Scheme 1:**
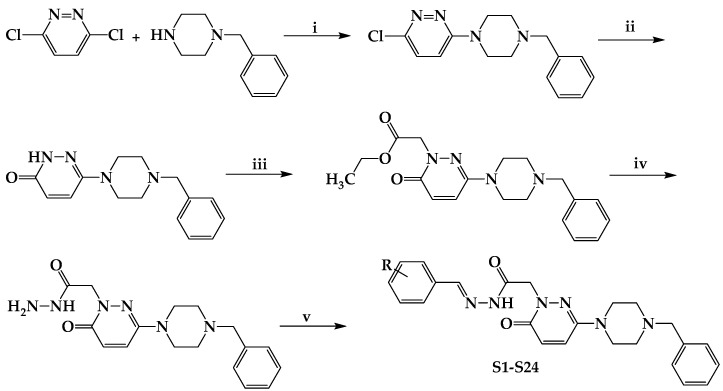
Synthesis and molecular structures of **S1**–**S24**. (i) EtOH, reflux for 6 h; (ii) AcOH, reflux for 6 h; (iii) BrCH_2_COOCH_2_CH_3_, K_2_CO_3_, acetone, reflux for 24 h; (iv) H_2_NNH_2_.H_2_O, MeOH, stirred for 3 h at rt; (v) suitable benzaldehyde; EtOH, reflux for 6 h. R; H, F, Br, Cl, CN, CH_3_, C_2_H_5_, OCH_3_, and N(CH_3_)_2_.

Scheme 1 shows the preparation of compounds **S1**–**S24**. All chemicals were obtained from commercial suppliers. Chloroform–methanol (90:10) was used as the mobile phase in Merck Kieselgel 60 F254 aluminum plates (Darmstadt, Germany), and the reactions were monitored using thin-layer chromatography. The spots were identified under 254 nm UV light. Melting points (mp) were measured without correction using a Thomas–Hoover capillary melting point device (Fredericksburg, VA, USA). An Avonce 600 Ultrashield^TM^ (Bruker, Rheinstetten, Germany) NMR spectrometer was used to record 1H- and 13C-NMR (300 MHz) spectra. Tetramethylsilane was used as an internal reference, and the compounds were dissolved in dimethyl sulfoxide (DMSO-d6) for NMR spectroscopy. The chemical shifts are represented as δ (ppm) values. The terms “singlet”, “doublet”, “triplet”, “quartet”, “multiplet”, and “doublet of doublet” were used to identify the splitting patterns. Using positive ion (ESI+) and negative ion (ESI−) electrospray ionization procedures, the HRMS spectra of the synthesized compounds were acquired from their solutions in methanol using the Waters LCT Premier XE UPLC/MS TOFF system (Milford, MA, USA) and MassLynx 4.1 software.

#### 4.1.1. Experimental Procedure for Synthesis

According to the literature procedure, 0.01 mol of 3,6-dichloropyridazine and 0.01 mol of 4-benzylpiperidine were mixed in 15 mL of ethanol under reflux and heated for 6 h to synthesize 3-chloro-6-(4-benzylpiperidine-1-yl)pyridazine. The reaction medium was added to ice water, and the filtered precipitate was refined by crystallizing ethanol. A solution of 0.05 mol of 3-chloro-6-(4-benzylpiperidine-1-yl)pyridazine in 30 mL glacial acetic acid was refluxed for 6 h to obtain 6-(4-benzylpiperidine-1-yl)-3(2H)-pyridazinone. Reduced pressure was used to remove the acetic acid, and the residue was dissolved in water and extracted using chloroform. After drying over sodium sulfate, the organic phase was evaporated at lower pressure. The residue was separated from ethanol using recrystallization. A total of 0.01 mol 6-(4-benzylpiperidine-1-yl)-3(2H)-pyridazinone, 0.02 mol ethyl bromoacetate, and 0.02 mol potassium carbonate in 40 mL of acetone were refluxed overnight to obtain ethyl 6-(4-benzylpiperidine-1-yl)-3(2H)-pyridazinone-2-ylacetate. After cooling, the solvent was evaporated, the organic salts were filtered, and the residue was refined using recrystallization from n-hexane to obtain the esters. To create 6-(4-benzylpiperidine-1-yl)-3(2H)-pyridazinone -2-ylacetohydrazide, 0.01 mol of ethyl 6-(4-benzylpiperidine-1-yl)-3(2H)-pyridazinone-2-yl acetate in 25 mL of methanol was combined with hydrazine hydrate (99%, 3 mL), and the mixture was stirred constantly for 3 h at room temperature. After filtering the precipitate, the ethanol was dried, cleaned with water, and recrystallized [15].

#### 4.1.2. Experimental Protocol for Producing the Chemicals Listed in the Title (**S1**–**S24**)

In 15 mL of ethanol, 0.01 mol of 6-(4-(4-benzylpiperidine-1-yl)-3(2H)-pyridazinone-2-yl acetohydrazide and 0.01 mol of substituted/nonsubstituted benzaldehyde were mixed and refluxed for 6 h. The mixture was then placed in frozen water to stop the reaction. The precipitate was filtered, dried, and crystallized in methanol–water (Scheme 1).

Eight of the compounds (**S1**, **S2**, **S6**, **S8**, **S11**, **S12**, **S13**, and **S14**) were created earlier, and their analgesic and anti-inflammatory properties were assessed before being published [15]. All the compounds’ spectrum data matched their given structures, as indicated below:

N′-2-bromobenzylidene-2-(3-(4-benzylpiperidin-1-yl)-6-oxopyridazin-1(6H)-yl)acetohydrazide (**S3**)

**S3** was obtained as a white solid by following a standard protocol with 6-(4-benzylpiperidine-1-yl)-3(2H)-pyridazinone-2-ylacetohydrazide and 2-bromobenzaldehyde. (m.p. 188–189, yield 66.39%). ^1^H-NMR (CDCl_3_, 300 MHz) ^TM^ 1.24–1.37 (2H, m, piperidine protons), 1.69–1.74 (3H, m, piperidine protons), 2.57 (d, 2H, benzyl CH_2_), 2.64–2.78 (4H piperidine protons), 5.27 (2H, s, CH_2_-CO-), 6.90–7.35 (m, 9H, phenyl protons), 7.57–7.60 (1H, d, pyridazinone H^5^), 7.95–7.98 (1H, d, pyridazinone H^4^), 8.18 (1H, s, N=CH), 9.44 (1H, s, CO-NH-N). ^13^C-NMR (CDCl_3_, 300 MHz), δ 31.37 (1C; CH_2_), 31.47 (1C; CH_2_), 37.82 (1C; CH), 43.04 (1C; CH_2_), 47.05 (2C; CH_2_-N), 53.58 (1C; N-CH_2_-C=O), 111.15 (1C; -CN), 125.97 (1C; N=CH), 127.04 (1C; pyridazinone C^5^), 128.27 (1C; pyridazinone C^4^), 130.18, 130.63, 130.74, 132.79, 132.88, 133.44 (6C; phenyl carbons), 139.72 (1C; 2-bromophenyl C^6^), 140.22 (1C; 2-bromophenyl C^5^) 143.68 (1C; 2-bromophenyl C^4^), 149.59 (1C; 2-bromophenyl C^1^), 150.07 (1C; 2-bromophenyl C^3^), 158.81 (1C; pyridazinone C^6^), 159.00 (1C; 2-bromophenyl C^2^), 164.54 (1C; -C=O) and 168.93 (1C; pyridazinone C^3^). C_25_H_26_BrN_5_O_2_ MS (ESI+) (Calc.: 508.1348; Found: 508.1352; 510.1351) (M^+^; 100.0%).

N′-3-bromobenzylidene-2-(3-(4-benzylpiperidin-1-yl)-6-oxopyridazin-1(6H)-yl)acetohydrazide (**S4**)

**S4** was obtained as a white solid by following standard protocol with 6-(4-benzylpiperidine-1-yl)-3(2H)-pyridazinone-2-ylacetohydrazide and 3-bromobenzaldehyde (m.p. 190–191, yield 69.67%). ^1^H-NMR (CDCl_3_, 300 MHz) ^TM^ 1.24–1.37 (2H, m, piperidine protons), 1.69–1.74 (2H, m, piperidine protons), 2.57 (d, 2H, benzyl CH_2_), 2.68–2.73 (1H, m, piperidine proton), 3.77–3.89 (4H piperidine protons), 5.27 (2H, s, CH_2_-CO-), 6.90–7.37 (m, 9H, phenyl protons), 7.56–7.60 (1H, d, pyridazinone H^5^), 7.95–7.98 (1H, d, pyridazinone H^4^), 8.18 (1H, s, N=CH), 9.44 (1H, s, CO-NH-N). ^13^C-NMR (CDCl_3_, 300 MHz), δ 31.36 (1C; CH_2_), 31.47 (1C; CH_2_), 37.80 (1C; CH), 43.03 (1C; CH_2_), 47.07 (2C; CH_2_-N), 53.46 (1C; N-CH_2_-C=O), 113.16 (1C; -CN), 126.01 (1C; N=CH), 128.29 (1C; pyridazinone C^5^), 129.63 (1C; pyridazinone C^4^), 130.38, 130.48, 130.66, 130.98, 131.10, 131.44 (6C; phenyl carbons), 134.98 (1C; 3-bromophenyl C^6^), 142.00 (1C; 3-bromophenyl C^5^), 146.18 (1C; 3-bromophenyl C^4^), 149.57 (1C; 3-bromophenyl C^1^), 150.14 (1C; 3-bromophenyl C^2^), 158.69 (1C; pyridazinone C^6^), 159.00 (1C; 3-bromophenyl C^3^), 164.38 (1C; -C=O) and 169.08 (1C; pyridazinone C^3^). C_25_H_26_BrN_5_O_2_ MS (ESI+) (Calc.: 508.4200; Found: 508.42) (M^+^; 100.0%).

N′-3-chlorobenzylidene-2-(3-(4-benzylpiperidin-1-yl)-6-oxopyridazin-1(6H)-yl)acetohydrazide (**S5**)

**S5** was obtained as a white solid by following standard protocol with 6-(4-benzylpiperidine-1-yl)-3(2H)-pyridazinone-2-ylacetohydrazide and 3-chlorobenzaldehyde (m.p. 137–138, yield 87.29%). ^1^H-NMR (CDCl_3_, 300 MHz) ^TM^ 1.2–1.32 (4H, m, piperidin protons), 2.56 (d, 2H, benzyl CH_2_), 2.69–2.73 (1H, m, piperidin proton), 3.74–3.85 (4H piperidin protons), 5.25 (2H, s, CH_2_-CO-), 7.17–7.35 (m, 9H, phenyl protons, pyridazinone H^5^), 7.5 (1H, d, pyridazinone H^4^), 7.78 (1H, s, 3-chlorophenyl H^2^), 7.64 (1H, s, N=CH), 10.05 (1H, s, CO-NH-N). ^13^C-NMR (CDCl_3_, 300 MHz), δ 31.36 (1C; CH_2_), 31.46 (1C; CH_2_), 37.80 (1C; CH), 43.04 (1C; CH_2_), 47.08 (2C; CH_2_-N), 53.34 (1C; N-CH_2_-C=O), 125.60 (1C; N=CH), 128.31 (1C; pyridazinone C^5^), 129.12 (1C; pyridazinone C^4^), 129.79, 129.97, 130.11, 130.29, 130.42, 130.70 (6C; phenyl carbons), 140.18 (1C; 3-chlorophenyl C^6^), 143.03 (1C; 3-chloroyphenyl C^5^) 147.31 (1C; 3-chlorophenyl C^2^), 149.51 (1C; 3-chlorophenyl C^1^), 150.08 (1C; 3-chlorophenyl C^4^), 158.72 (1C; pyridazinone C^6^), 159.01 (1C; 3-chlorophenyl C^3^), 164.29 (1C; -C=O) and 169.07 (1C; pyridazinone C^3^). C_25_H_26_ClN_5_O_2_ MS (ESI+) (Calc.: 464.1353; Found: 464.1353) (M^+^; 100.0%).

N′-3-fluorobenzylidene-2-(3-(4-benzylpiperidin-1-yl)-6-oxopyridazin-1(6H)-yl)acetohydrazide (**S7**)

**S7** was obtained as a white solid by following standard protocol with 6-(4-benzylpiperidine-1-yl)-3(2H)-pyridazinone-2-ylacetohydrazide and 3-fluorobenzaldehyde (m.p. 197–198, yield 92.33%). ^1^H-NMR (CDCl_3_, 300 MHz) ^TM^ 1.23–1.32 (3H, m, piperidin protons), 2.55–2.57 (d, 2H, benzyl CH_2_), 2.69–2.74 (2H, m, piperidin proton), 3.74–3.86 (4H piperidin protons), 5.25 (2H, s, CH_2_-CO-), 7.14–7.39 (m, 11H, phenyl protons, pyridazinone H^4,5^), 7.78 (1H, s, N=CH), 9.87 (1H, s, CO-NH-N). ^13^C-NMR (CDCl_3_, 300 MHz), δ 31.36 (1C; CH_2_), 31.47 (1C; CH_2_), 37.81 (1C; CH), 43.04 (1C; CH_2_), 47.08 (2C; CH_2_-N), 53.32 (1C; N-CH_2_-C=O), 113.20 (1C; N=CH), 117.25 (1C; pyridazinone C^5^), 128.28 (1C; pyridazinone C^4^), 129.12, 130.13, 130.29, 130.34, 130.43, 130.72 (6C; phenyl carbons), 140.19 (1C; 3-fluorophenyl C^6^), 143.11 (1C; 3-fluoroyphenyl C^5^), 147.56 (1C; 3-fluorophenyl C^2^), 149.49 (1C; 3-fluorophenyl C^1^), 150.08 (1C; 3-fluorophenyl C^4^), 158.70 (1C; pyridazinone C^6^), 163.81 (1C; 3-fluorophenyl C^3^), 164.30 (1C; -C=O) and 168.97 (1C; pyridazinone C^3^). C_25_H_26_FN_5_O_2_ MS (ESI+) (Calc.: 448.2149; Found: 448.2147) (M^+^; 100.0%).

N′-2-methylbenzylidene-2-(3-(4-benzylpiperidin-1-yl)-6-oxopyridazin-1(6H)-yl)acetohydrazide (**S9**)

**S9** was obtained as a white solid by following standard protocol with 6-(4-benzylpiperidine-1-yl)-3(2H)-pyridazinone-2-ylacetohydrazide and 2-methylbenzaldehyde (m.p. 222–223, yield 43.56%). ^1^H-NMR (CDCl_3_, 300 MHz) ^TM^ 1.26–1.74 (5H, m, piperidin protons), 2.44 (s, 3H, CH_3_), 2.71 (d, 2H, benzyl CH_2_), 3.76–3.85 (4H piperidin protons), 4.82 (2H, s, CH_2_-CO-), 6.88–7.72 (m, 9H, phenyl protons), 7.73 (1H, d, pyridazinone H^5^), 7.94 (1H, d, pyridazinone H^4^), 8.32 (1H, s, N=CH), 10.59 (1H, s, CO-NH-N). ^13^C-NMR (CDCl_3_, 300 MHz), δ 20.16 (1C; CH_3_), 31.37 (1C; CH_2_), 31.49 (1C; CH_2_), 37.82 (1C; CH), 43.05 (1C; CH_2_), 47.10 (2C; CH_2_-N), 53.33 (1C; N-CH_2_-C=O), 125.98 (1C; N=CH), 126.25 (1C; pyridazinone C^5^), 127.49 (1C; pyridazinone C^4^), 129.97, 130.20, 130.48, 130.69, 130.75, 131.11 (6C; phenyl carbons), 131.62, 137.10, 140.21, 143.40, 147.35, 149.42 (6C; 2-methylphenyl carbons), 150.04 (1C; pyridazinone C^6^), 158.71 (1C; -C=O) and 168.54 (1C; pyridazinone C^3^). C_26_H_29_N_5_O_2_ MS (ESI+) (Calc.: 444.2400; Found: 444.2409) (M^+^; 100.0%).

N′-3-methylbenzylidene-2-(3-(4-benzylpiperidin-1-yl)-6-oxopyridazin-1(6H)-yl)acetohydrazide (**S10**)

**S10** was obtained as a white solid by following standard protocol with 6-(4-benzylpiperidine-1-yl)-3(2H)-pyridazinone-2-ylacetohydrazide and 3-methylbenzaldehyde (m.p. 106–107, yield 86.98%). ^1^H-NMR (CDCl_3_, 300 MHz) ^TM^ 1.23–1.32 (2H, m, piperidin protons), 2.35 (s, 3H, CH_3_), 2.55 (2H, d, benzyl CH_2_), 2.69–2.73 (3H piperidin protons), 3.74–3.86 (4H piperidin protons), 5.27 (2H, s, CH_2_-CO-), 7.21–7.30 (m, 9H, phenyl protons, pyridazinone H^5^), 7.44 (1H, s, 3-methylphenyl C^2^), 7.47 (1H, d, pyridazinone H^4^), 7.74 (1H, s, N=CH), 9.57 (1H, s, CO-NH-N). ^13^C-NMR (CDCl_3_, 300 MHz), δ 21.34 (1C; CH_3_), 31.36 (1C; CH_2_), 31.48 (1C; CH_2_), 37.81 (1C; CH), 43.05 (1C; CH_2_), 47.10 (2C; CH_2_-N), 53.28 (1C; N-CH_2_-C=O), 124.67 (1C; N=CH), 125.41 (1C; pyridazinone C^5^), 127.64 (1C; pyridazinone C^4^), 128.27, 128.62, 129.12, 130.47, 130.74, 131.12 (6C; phenyl carbons), 131.35, 138.45, 144.61, 149.12, 149.45, 150.03, (6C; 3-methylphenyl carbons), 159.06 (1C; pyridazinone C^6^), 164.20 (1C; -C=O) and 168.80 (1C; pyridazinone C^3^). C_26_H_29_N_5_O_2_ MS (ESI+) (Calc.: 444.2400; Found: 444.2419) (M^+^; 100.0%).

N′-4-ethylbenzylidene-2-(3-(4-benzylpiperidin-1-yl)-6-oxopyridazin-1(6H)-yl)acetohydrazide (**S15**)

**S15** was obtained as a white solid by following standard protocol with 6-(4-benzylpiperidine-1-yl)-3(2H)-pyridazinone-2-ylacetohydrazide and 4-ethylbenzaldehyde (m.p. 225–226, yield 78.62%). ^1^H-NMR (CDCl_3_, 300 MHz) ^TM^ 1.24–1.29 (3H, t, CH_3_), 1.28–1.32 (2H, q, -CH_2_-), 1.71–1.72 (3H, m, piperidin proton), 2.58 (d, 2H, benzyl CH_2_), 2.66–2.74 (2H, m, piperidin proton), 3.78–3.89 (4H piperidin protons), 5.26 (2H, s, CH_2_-CO-), 7.14–7.30 (m, 9H, phenyl protons), 7.75–7.78 (1H, d, pyridazinone H^5^), 7.82–7.85 (1H, d, pyridazinone H^4^), 8.12 (1H, s, N=CH), 10.96 (1H, s, CO-NH-N). ^13^C-NMR (CDCl_3_, 300 MHz), δ 15.35 (1C; CH_3_), 28.85 (1C; CH_2_), 31.36 (1C; CH_2_), 31.49 (1C; CH_2_), 37.82 (1C; CH), 43.05 (1C; CH_2_), 47.10 (2C; CH_2_-N), 53.24 (1C; N-CH_2_-C=O), 125.97 (1C; N=CH), 126.28 (1C; pyridazinone C^5^), 127.32 (1C; pyridazinone C^4^), 127.87, 128.14, 128.31, 129.12, 130.76, 131.01 (6C; phenyl carbons), 144.36, 146.93, 149.07, 149.71, 150.02, 158.70 (6C; 4-methylphenyl carbons), 159.07 (1C; pyridazinone C^6^), 164.15 (1C; -C=O) and 168.61 (1C; pyridazinone C^3^). C_27_H_31_N_5_O_2_ MS (ESI+) (Calc.: 458.2556; Found: 458.2556) (M^+^; 100.0%).

N′-2-cyanobenzylidene-2-(3-(4-benzylpiperidin-1-yl)-6-oxopyridazin-1(6H)-yl)acetohydrazide (**S16**)

**S16** was obtained as a white solid by following standard protocol with 6-(4-benzylpiperidine-1-yl)-3(2H)-pyridazinone-2-ylacetohydrazide and 2-cyanobenzaldehyde (m.p. 189–190, yield 66.85%). ^1^H-NMR (CDCl_3_, 300 MHz) ^TM^ 1.21–1.29 (2H, m, piperidin protons), 1.67–1.75 (2H, m, piperidin protons), 2.56 (d, 2H, benzyl CH_2_), 2.69 (1H, m, piperidin proton), 3.76–3.80 (4H piperidin protons), 5.25 (2H, s, CH_2_-CO-), 7.18–7.38 (m, 9H, phenyl protons), 7.87–7.89 (1H, d, pyridazinone H^5^), 8.06 (1H, d, pyridazinone H^4^), 8.43 (1H, s, N=CH), 10.50 (1H, s, CO-NH-N). ^13^C-NMR (CDCl_3_, 300 MHz), δ 31.37 (1C; CH_2_), 31.47 (1C; CH_2_), 37.82 (1C; CH), 43.04 (1C; CH_2_), 47.05 (2C; CH_2_-N), 53.58 (1C; N-CH_2_-C=O), 111.15 (1C; -CN), 125.97 (1C; N=CH), 127.04 (1C; pyridazinone C^5^), 128.27 (1C; pyridazinone C^4^), 130.18, 130.63, 130.74, 132.79, 132.88, 133.44 (6C; phenyl carbons), 139.72 (1C; 2-cyanophenyl C^6^), 140.22 (1C; 2-cyanophenyl C^5^) 143.68 (1C; 2-cyanophenyl C^4^), 149.59 (1C; 2-cyanophenyl C^1^), 150.07 (1C; 2-cyanophenyl C^3^), 158.81 (1C; pyridazinone C^6^), 159.00 (1C; 2-cyanophenyl C^2^), 164.54 (1C; -C=O) and 168.93 (1C; pyridazinone C^3^). C_26_H_26_N_6_O_2_ MS (ESI+) (Calc.: 455.2195; Found: 455.2191) (M^+^; 100.0%).

N′-3-cyanobenzylidene-2-(3-(4-benzylpiperidin-1-yl)-6-oxopyridazin-1(6H)-yl)acetohydrazide (**S17**)

**S17** was obtained as a white solid by following standard protocol with 6-(4-benzylpiperidine-1-yl)-3(2H)-pyridazinone-2-ylacetohydrazide and 3-cyanobenzaldehyde (m.p. 128–129, yield 86.49%). ^1^H-NMR (CDCl_3_, 300 MHz) ^TM^ 1.25–1.33 (4H, m, piperidin protons), 2.55 (d, 2H, benzyl CH_2_), 2.69 (1H, m, piperidin proton), 3.76–3.85 (4H piperidin protons), 5.25 (2H, s, CH_2_-CO-), 6.85–6.91 (m, 9H, phenyl protons), 7.21 (1H, s, 3-cyanophenyl H^2^), 7.28 (1H, d, pyridazinone H^5^), 7.69 (1H, d, pyridazinone H^4^), 7.98 (1H, s, N=CH), 10.67 (1H, s, CO-NH-N). ^13^C-NMR (CDCl_3_, 300 MHz), δ 31.36 (1C; CH_2_), 31.47 (1C; CH_2_), 37.80 (1C; CH), 43.03 (1C; CH_2_), 47.07 (2C; CH_2_-N), 53.46 (1C; N-CH_2_-C=O), 113.16 (1C; -CN), 126.01 (1C; N=CH), 128.29 (1C; pyridazinone C^5^), 129.63 (1C; pyridazinone C^4^), 130.38, 130.48, 130.66, 130.98, 131.10, 131.44 (6C; phenyl carbons), 134.98 (1C; 3-cyanophenyl C^6^), 142.00 (1C; 3-cyanophenyl C^5^), 146.18 (1C; 3-cyanophenyl C^4^), 149.57 (1C; 3-cyanophenyl C^1^), 150.14 (1C; 3-cyanophenyl C^2^), 158.69 (1C; pyridazinone C^6^), 159.00 (1C; 3-cyanophenyl C^3^), 164.38 (1C; -C=O) and 169.08 (1C; pyridazinone C^3^). C_26_H_26_N_6_O_2_ MS (ESI+) (Calc.: 455.2195; Found: 455.2200) (M^+^; 100.0%).

N′-4-cyanobenzylidene-2-(3-(4-benzylpiperidin-1-yl)-6-oxopyridazin-1(6H)-yl)acetohydrazide (**S18**)

**S18** was obtained as a white solid by following standard protocol with 6-(4-benzylpiperidine-1-yl)-3(2H)-pyridazinone-2-ylacetohydrazide and 4-cyanobenzaldehyde (m.p. 129–130, yield 69.47%). ^1^H-NMR (CDCl_3_, 300 MHz) ^TM^ 1.25–1.33 (2H, m, piperidin protons), 1.62–1.72 (2H, m, piperidin protons), 2.57 (d, 2H, benzyl CH_2_), 2.66–2.75 (1H, m, piperidin proton), 3.77–3.87 (4H piperidin protons), 5.28 (2H, s, CH_2_-CO-), 7.15–7.32 (m, 9H, phenyl protons), 7.73 (1H, d, pyridazinone H^5^), 8.01 (1H, d, pyridazinone H^4^), 9.74 (1H, s, N=CH), 10.73 (1H, s, CO-NH-N). ^13^C-NMR (CDCl_3_, 300 MHz), δ 31.36 (1C; CH_2_), 31.47 (1C; CH_2_), 37.82 (1C; CH), 43.03 (1C; CH_2_), 47.06 (2C; CH_2_-N), 53.23 (1C; N-CH_2_-C=O), 104.88 (1C; -CN), 126.05 (1C; N=CH), 128.28 (1C; pyridazinone C^5^), 129.11 (1C; pyridazinone C^4^), 130.43, 130.71, 140.04, 140.15, 144.38 (6C; phenyl carbons), 149.05 (1C; 4-cyanophenyl C^6^), 149.48 (1C; 4-cyanophenyl C^2^), 150.04 (1C; 4-cyanophenyl C^1^), 153.37 (1C; 4-cyanophenyl C^3^), 153.49 (1C; 4-cyanophenyl C^5^), 158.67 (1C; pyridazinone C^6^), 159.04 (1C; 4-cyanophenyl C^4^), 164.17 (1C; -C=O) and 168.74 (1C; pyridazinone C^3^). C_26_H_26_N_6_O_2_ MS (ESI+) (Calc.: 455.2195; Found: 455.2202) (M^+^; 100.0%).

N′-2-methoxybenzylidene-2-(3-(4-benzylpiperidin-1-yl)-6-oxopyridazin-1(6H)-yl)acetohydrazide (**S19**)

**S19** was obtained as a white solid by following standard protocol with 6-(4-benzylpiperidine-1-yl)-3(2H)-pyridazinone-2-ylacetohydrazide and 2-methoxybenzaldehyde (m.p. 206–207, yield 92.37%). ^1^H-NMR (CDCl_3_, 300 MHz) ^TM^ 1.23–1.33 (2H, m, piperidin protons), 1.65–1.75 (2H, m, piperidin protons), 2.56 (d, 2H, benzyl CH_2_), 2.64–2.74 (1H, m, piperidin proton), 3.74–3.79 (4H piperidin protons), 3.86 (3H, s, OCH_3_), 5.25 (2H, s, CH_2_-CO-), 7.12–7.30 (m, 9H, phenyl protons), 7.47–7.49 (1H, d, pyridazinone H^5^), 7.65 (1H, d, pyridazinone H^4^), 8.36 (1H, s, N=CH), 10.53 (1H, s, CO-NH-N). ^13^C-NMR (CDCl_3_, 300 MHz), δ 31.35 (1C; CH_2_), 31.49 (1C; CH_2_), 37.83 (1C; CH), 43.05 (1C; CH_2_), 47.11 (2C; CH_2_-N), 53.26 (1C; N-CH_2_-C=O), 55.56 (1C; OCH_3_), 120.80 (1C; N=CH), 125.97 (1C; pyridazinone C^5^), 126.49 (1C; pyridazinone C^4^), 127.18, 128.27, 128.31, 129.12, 130.54, 130.76 (6C; phenyl carbons), 131.58 (1C; 2-methoxyphenyl C^6^), 131.78 (1C; 2-methoxyphenyl C^5^) 140.06 (1C; 2-methoxyphenyl C^4^), 144.78 (1C; 2-methoxyphenyl C^1^), 149.99 (1C; 2-methoxyphenyl C^3^), 158.14 (1C; pyridazinone C^6^), 159.07 (1C; 2-methoxyphenyl C^2^), 164.14 (1C; -C=O) and 168.27 (1C; pyridazinone C^3^). C_26_H_29_N_5_O_3_ MS (ESI+) (Calc.: 460.2349; Found: 460.2352) (M^+^; 100.0%).

N′-3,5-dimethoxybenzylidene-2-(3-(4-benzylpiperidin-1-yl)-6-oxopyridazin-1(6H)-yl)acetohydrazide (**S20**)

**S20** was obtained as a white solid by following standard protocol with 6-(4-benzylpiperidine-1-yl)-3(2H)-pyridazinone-2-ylacetohydrazide and 3,5-dimethoxybenzaldehyde (m.p. 208–209, yield 97.37%). ^1^H-NMR (CDCl_3_, 300 MHz) ^TM^ 1.25–1.32 (4H, m, piperidin protons), 2.56 (d, 2H, benzyl CH_2_), 2.64–2.72 (1H, m, piperidin proton), 3.75–3.76 (2H, m, piperidin protons), 3.78 (2H, m, piperidin protons), 3.84 (3H, s, OCH_3_), 3.88 (3H, s, OCH_3_), 5.22 (2H, s, CH_2_-CO-), 7.18–7.34 (m, 9H, phenyl protons, pyridazinone H^4,5^), 8.03 (1H, s, 3,5-dimethoxyphenyl H^4^), 8.52 (1H, s, N=CH), 10.36 (1H, s, CO-NH-N). ^13^C-NMR (CDCl_3_, 300 MHz), δ 31.35 (1C; CH_2_), 31.47 (1C; CH_2_), 37.80 (1C; CH), 43.04 (1C; CH_2_), 47.07 (2C; CH_2_-N), 53.21 (1C; N-CH_2_-C=O), 55.38 (1C; OCH_3_), 55.47 (1C; OCH_3_), 104.56 (1C; N=CH), 125.98 (1C; pyridazinone C^5^), 126.04 (1C; pyridazinone C^4^), 126.31, 126.57, 128.27, 129.12, 130.73, 135.41 (6C; phenyl carbons), 140.20 (1C; 3,5-dimethoxyphenyl C^6^), 141.56 (1C; 3,5-dimethoxyphenyl C^2^), 144.28 (1C; 3,5-dimethoxyphenyl C^1^), 149.06 (1C; 3,5-dimethoxyphenyl C^4^), 149.44 (1C; pyridazinone C^6^), 158.67 (1C; 3,5-dimethoxyphenyl C^5^), 160.99 (1C; 3,5-dimethoxyphenyl C^3^), 164.25 (1C; -C=O) and 168.76 (1C; pyridazinone C^3^). C_27_H_31_N_5_O_4_ MS (ESI+) (Calc.: 490.2454; Found: 490.2461) (M^+^; 100.0%).

N′-2,4,6-trimethoxybenzylidene-2-(3-(4-benzylpiperidin-1-yl)-6-oxopyridazin-1(6H)-yl)acetohydrazide (**S21**)

**S21** was obtained as a white solid by following standard protocol with 6-(4-benzylpiperidine-1-yl)-3(2H)-pyridazinone-2-ylacetohydrazide and 2,4,6-trimethoxybenzaldehyde (m.p. 160–161, yield 54.81%). ^1^H-NMR (CDCl_3_, 300 MHz) ^TM^ 1.20–1.32 (2H, m, piperidin protons), 1.65–1.75 (2H, m, piperidin protons), 2.56 (d, 2H, benzyl CH_2_), 2.66–2.67 (1H, m, piperidin proton), 3.80 (4H piperidin protons), 3.82 (3H, s, OCH_3_), 3.84 (6H, s, OCH_3_), 5.23 (2H, s, CH_2_-CO-), 7.17–7.30 (m, 10H, phenyl protons, pyridazinone H^4,5^), 8.02 (1H, s, 2,4,6-trimethoxyphenyl H^2^), 8.33 (1H, s, N=CH), 10.29 (1H, s, CO-NH-N). ^13^C-NMR (CDCl_3_, 300 MHz), δ 31.36 (1C; CH_2_), 31.47 (1C; CH_2_), 37.79 (1C; CH), 43.03 (1C; CH_2_), 47.06 (2C; CH_2_-N), 53.23 (1C; N-CH_2_-C=O), 56.11 (1C; OCH_3_), 56.21 (1C; OCH_3_), 56.26 (1C; OCH_3_), 104.88 (1C; N=CH), 126.00 (1C; pyridazinone C^5^), 126.05 (1C; pyridazinone C^4^), 126.32, 128.28, 129.04, 129.11, 130.43, 130.71 (6C; phenyl carbons), 140.15 (1C; 2,4,6-trimethoxyphenyl C^5^), 144.38 (1C; 2,4,6-trimethoxyphenyl C^1^), 149.48 (1C; 2,4,6-trimethoxyphenyl C^3^), 150.04 (1C; pyridazinone C^6^), 153.37 (1C; 2,4,6-trimethoxyphenyl C^2^), 153.49 (1C; 2,4,6-trimethoxyphenyl C^4^), 159.04 (1C; 2,4,6-trimethoxyphenyl C^6^), 164.17 (1C; -C=O) and 168.74 (1C; pyridazinone C^3^). C_28_H_33_N_5_O_5_ MS (ESI+) (Calc.: 520.2560; Found: 520.2563) (M^+^; 100.0%).

N′-2,3-dimethoxybenzylidene-2-(3-(4-benzylpiperidin-1-yl)-6-oxopyridazin-1(6H)-yl)acetohydrazide (**S22**)

**S22** was obtained as a white solid by following standard protocol with 6-(4-benzylpiperidine-1-yl)-3(2H)-pyridazinone-2-ylacetohydrazide and 2,3-dimethoxybenzaldehyde (m.p. 212–213, yield 76.95%). ^1^H-NMR (CDCl_3_, 300 MHz) ^TM^ 1.24–1.31 (4H, m, piperidin protons), 1.68–1.72 (1H, m, piperidin protons), 2.55–2.71 (4H, benzyl CH_2_, piperidin proton), 3.75–3.77 (3H, m, piperidin protons), 3.86 (3H, s, OCH_3_), 3.89 (3H, s, OCH_3_), 5.25 (2H, s, CH_2_-CO-), 6.88–7.48 (m, 10H, phenyl protons, pyridazinone H^4,5^), 8.09 (1H, s, N=CH), 9.06 (1H, s, CO-NH-N). ^13^C-NMR (CDCl_3_, 300 MHz), δ 31.35 (1C; CH_2_), 31.48 (1C; CH_2_), 37.81 (1C; CH), 43.04 (1C; CH_2_), 47.09 (2C; CH_2_-N), 53.27 (1C; N-CH_2_-C=O), 55.79 (1C; OCH_3_), 55.84 (1C; OCH_3_), 113.86 (1C; N=CH), 124.13 (1C; pyridazinone C^5^), 124.30 (1C; pyridazinone C^4^), 126.28, 126.53, 127.25, 128.26, 129.11, 130.73 (6C; phenyl carbons), 140.05 (1C; 2,3-dimethoxyphenyl C^6^), 144.49 (1C; 2,3-dimethoxyphenyl C^5^), 148.55 (1C; 2,3-dimethoxyphenyl C^1^), 149.40 (1C; 2,3-dimethoxyphenyl C^4^), 152.83 (1C; pyridazinone C^6^), 158.69 (1C; 2,3-dimethoxyphenyl C^2^), 159.03 (1C; 2,3-dimethoxyphenyl C^3^), 164.23 (1C; -C=O) and 168.40 (1C; pyridazinone C^3^). C_27_H_31_N_5_O_4_ MS (ESI+) (Calc.: 490.2454; Found: 490.2457) (M^+^; 100.0%).

N′-3,4,5-trimethoxybenzylidene-2-(3-(4-benzylpiperidin-1-yl)-6-oxopyridazin-1(6H)-yl)acetohydrazide (**S23**)

**S23** was obtained as a white solid by following standard protocol with 6-(4-benzylpiperidine-1-yl)-3(2H)-pyridazinone-2-ylacetohydrazide and 3,4,5-trimethoxybenzaldehyde (m.p. 146–147, yield 86.16%). ^1^H-NMR (CDCl_3_, 300 MHz) ^TM^ 1.27–1.71 (4H, m, piperidin protons), 2.55–2.69 (4H, benzyl CH_2_, piperidin proton), 3.78–3.81 (3H piperidin protons), 3.85 (9H, s, OCH_3_), 5.28 (2H, s, CH_2_-CO-), 6.89–7.32 (m, 10H, phenyl protons, pyridazinone H^4,5^), 7.30 (1H, s, 3,4,5-trimethoxyphenyl H^2^), 8.03 (1H, s, N=CH), 10.36 (1H, s, CO-NH-N). ^13^C-NMR (CDCl_3_, 300 MHz), δ 31.36 (1C; CH_2_), 31.47 (1C; CH_2_), 37.79 (1C; CH), 43.03 (1C; CH_2_), 47.06 (2C; CH_2_-N), 53.23 (1C; N-CH_2_-C=O), 56.11 (1C; OCH_3_), 56.21 (1C; OCH_3_), 56.26 (1C; OCH_3_), 104.88 (1C; N=CH), 126.00 (1C; pyridazinone C^5^), 126.05 (1C; pyridazinone C^4^), 126.32, 128.28, 129.04, 129.11, 130.43, 130.71 (6C; phenyl carbons), 140.15 (1C; 3,4,5-trimethoxyphenyl C^2^), 144.38 (1C; 3,4,5-trimethoxyphenyl C^1^), 149.48 (1C; 3,4,5-trimethoxyphenyl C^6^), 150.04 (1C; pyridazinone C^6^), 153.37 (1C; 3,4,5-trimethoxyphenyl C^3^), 153.49 (1C; 3,4,5-trimethoxyphenyl C^4^), 159.04 (1C; 3,4,5-trimethoxyphenyl C^5^), 164.17 (1C; -C=O) and 168.74 (1C; pyridazinone C^3^). C_28_H_33_N_5_O_5_ MS (ESI+) (Calc.: 520.2560; Found: 520.2558) (M^+^; 100.0%).

N′-2,4-dimethoxybenzylidene-2-(3-(4-benzylpiperidin-1-yl)-6-oxopyridazin-1(6H)-yl)acetohydrazide (**S24**)

**S24** was obtained as a white solid by following standard protocol with 6-(4-benzylpiperidine-1-yl)-3(2H)-pyridazinone-2-ylacetohydrazide and 2,4-dimethoxybenzaldehyde (m.p. 174–175, yield 61.26%). ^1^H-NMR (CDCl_3_, 300 MHz) ^TM^ 1.28–1.30 (4H, m, piperidin protons), 2.68 (d, 4H, benzyl CH_2_, piperidin proton), 3.76–3.80 (3H, m, piperidin protons), 3.88 (3H, s, OCH_3_), 3.90 (3H, s, OCH_3_), 4.81 (2H, s, CH_2_-CO-), 6.39–7.99 (m, 9H, phenyl protons, pyridazinone H^4,5^), 8.05 (1H, s, 2,4-dimethoxyphenyl H^4^), 8.33 (1H, s, N=CH), 10.29 (1H, s, CO-NH-N). ^13^C-NMR (CDCl_3_, 300 MHz), δ 31.35 (1C; CH_2_), 31.47 (1C; CH_2_), 37.80 (1C; CH), 43.04 (1C; CH_2_), 47.07 (2C; CH_2_-N), 53.21 (1C; N-CH_2_-C=O), 55.38 (1C; OCH_3_), 55.47 (1C; OCH_3_), 104.56 (1C; N=CH), 125.98 (1C; pyridazinone C^5^), 126.04 (1C; pyridazinone C^4^), 126.31, 126.57, 128.27, 129.12, 130.73, 135.41 (6C; phenyl carbons), 140.20 (1C; 2,4-dimethoxyphenyl C^6^), 141.56 (1C; 2,4-dimethoxyphenyl C^3^), 144.28 (1C; 2,4-dimethoxyphenyl C^1^), 149.06 (1C; 2,4-dimethoxyphenyl C^5^), 149.44 (1C; pyridazinone C^6^), 158.67 (1C; 2,4-dimethoxyphenyl C^2^), 160.99 (1C; 2,4-dimethoxyphenyl C^4^), 164.25 (1C; -C=O) and 168.76 (1C; pyridazinone C^3^). C_27_H_31_N_5_O_4_ MS (ESI+) (Calc.: 490.2454; Found: 490.2448) (M^+^; 100.0%).

### 4.2. Enzyme Inhibition Studies

#### 4.2.1. Enzyme Assays and Kinetics

The inhibition of hMAO-A and hMAO-B (recombinant; Sigma-Aldrich, St. Louis, MO, USA) was evaluated using 0.06 mM kynuramine and 0.3 mM benzylamine, respectively [34]. The absorbance of the MAO-A and MAO-B reactions was measured using the continuous method at 250 nm and 316 nm, respectively, for 45 min [34] with slight modifications. In the kinetic study, a Lineweaver–Burk (LB) plot was generated using five substrate concentrations, and the K_m_ value of benzylamine for MAO-B was calculated as 0.29 mM.

#### 4.2.2. An Inhibition Study of MAO-A and MAO-B by the Compounds

In the primary screening, 24 compounds were evaluated for their inhibition of MAO-A or MAO-B at 10 µM concentration. The half-inhibitory concentrations (IC_50_) of the compounds were calculated using GraphPad Prism software 5 [35]. The compounds with IC_50_ values of 40 µM or higher were indicated as >40 µM. The selectivity index (SI) was calculated as the IC_50_ of MAO-A/IC_50_ of MAO-B. The inhibition of compounds was compared with reference inhibitors such as toloxatone and clorgyline (reversible and irreversible inhibitors, respectively) for MAO-A and safinamide and pargyline (reversible and irreversible inhibitors, respectively) for MAO-B inhibitors [35]. For the inhibition kinetics, lead compounds were used at three concentrations, approximately 0.5, 1.5, and 2.0 times the IC_50_ value [34], and the inhibition constant (K_i_) was determined using the secondary plot of their slopes in the LB plots.

#### 4.2.3. Reversibility Studies

The reversibility of the lead compounds to MAO-B was evaluated using a dialysis tube (D-Tube Dialyzer Maxi, MWCO 6–8 kDa, Sigma-Aldrich, St. Louis, MO, USA). Their reversibilities were analyzed by the values of undialyzed (A_U_) and dialyzed (A_D_) activities, measuring approximately 2.0 times the IC_50_ value after pre-incubation for 30 min [34]. The reversibility patterns of the lead compounds were determined by comparing A_U_ and A_D_ with those reported in the literature [34].

### 4.3. Blood–Brain Barrier Permeability Study

In early drug studies, a parallel artificial membrane permeation approach was employed to predict the passive transcellular permeability of a drug through the blood–brain barrier (BBB). This involved setting up a sandwich-like structure in the PAMPA using a microtiter plate with 96 wells and a Millipore filter plate with 96 wells (IPVH, 125 m thick filter, 0.45 m pore), which were then submerged in 0.1 mL of n-dodecane. Drug samples were initially prepared as stock solutions in DMSO at 10 mm doses and stored at 0 °C. Before being introduced into a 96-well filter plate, these stock solutions were further diluted at pH 7.4 to achieve final sample concentrations of 0.01, 0.1, 0.5, and 1 mM, with the DMSO content limited to 1% (*v*/*v*). The resulting diluted solutions were then transferred to the donor wells (270 µL each), with 200 µL of pH 7.4 buffering agent added to the acceptor well. To create the “sandwich”, the donor plate (with the analyte at the bottom, an aqueous recipient on top, and an artificial lipid barrier in the middle) was accurately positioned over the acceptor filter plate. The drug material diffused from the donor well into the acceptor well through the lipid membrane, with the “sandwich” structure remaining intact throughout the process. UV spectroscopy was used to measure the drug concentration in the donor, recipient, and reference wells, and the extent of permeation was assessed using a specific expression [36].

Log Pe=−ln[1−CA/Equilibrium]/A×(1/VD+1/VA)×t

$$Where,Pe\;is\;permeability\;(cm\cdot s^{-1}),$$

CA=receptor concentration,

$$A=area\;of\;effective\;filtration\;(4.8\;{cm}^{2}),$$

VD=donor volume (mL),

VA=acceptor volume (mL),

t=time of incubation (s) and

Equilibrium=(CD×VD+CA×VA)/(VD+VA)

### 4.4. Molecular Docking

The molecular docking software AutoDock Vina and the PDBQT molecular structure file format were used [37]. Consequently, AutoDockTools-1.5.6, from the MGLTools-1.5.6 package, was used to create ligands and receptors in the PDBQT file format. ChemDraw 23 was used to draw the **S5** chemical structure in pdf format. Furthermore, Chembio3D pro was utilized for optimization, for improved molecular confirmation, using energy minimization with a 0.01 RMS gradient. Three-dimensional ligands were corrected by substituting hydrogen atoms in the water molecules. The crystal structure of 2V5Z was obtained from the Protein Data Bank. Following the extraction of the co-crystallized ligands, ions, and water molecules, a 40 × 40 × 40 grid box with a grid spacing of 0.375, X = 51.901, Y = 156.468, and Z = 28.561 was formed. The receptor file [38] was saved in the PDBQT file format. Vina was run on a Windows 10 PC with a Core i5 processor. A standard configuration file was established to specify grid box coordinates, exhaustiveness, computer usage, and output data for the docking experiment. The search was set to eight intensities to cover as much of the ground as possible. The docking experiments were initiated using a command line.

The docking experiment produced a PDBQT file with 20 different docked ligand positions on the receptor. The ideal position was selected by considering the docking score, number of H-bonds, and visual inspection of each docking position. The PDB format was used to store the selected receptor and ligand positions. The resulting PDB file was examined for ligand–receptor interactions using LigPlot + version 2.2 [39,40] and Biovia Discovery Studio 2021.

## 5. Conclusions

Twenty-four derivatives of pyridazinobenzylpiperidine were prepared, and their ability to inhibit monoamine oxidase (MAO) was assessed. The compound displayed greater MAO-B than MAO-A inhibition. Compound **S15** demonstrated the most potent MAO-A inhibition, whereas compounds **S5** and **S16** demonstrated significant competitive and reversible MAO-B inhibition. Overall, if we look into the selectivity index, we can conclude that most of the compounds showed non-specific MAO inhibition. Additionally, BBB penetration was demonstrated by compounds **S5** and **S16**, and molecular docking experiments indicated that compound **S5** stabilized the protein–ligand complex. Based on these findings, lead compounds may be used as medications to treat neurological conditions.

## Figures and Tables

**Table 1:** Structures, yields, and chemical–physical data of titled compounds.

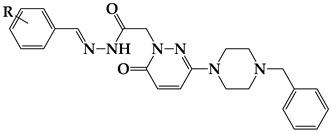					
Compound	R	Yield (%)	M.P. (°C)	M.W.	Molecular Formula
**S1**	H	30.45	210–211	429.23	C_25_H_27_N_5_O_2_
**S2**	4-Br	58.08	189–190	507.13	C_25_H_26_BrN_5_O_2_
**S3**	2-Br	66.39	188–189	507.13	C_25_H_26_BrN_5_O_2_
**S4**	3-Br	69.67	190–191	507.13	C_25_H_26_BrN_5_O_2_
**S5**	3-Cl	87.29	137–138	464.14	C_25_H_26_ClN_5_O_2_
**S6**	4-Cl	69.67	240–241	464.14	C_25_H_26_ClN_5_O_2_
**S7**	3-F	92.33	197–198	448.21	C_25_H_26_FN_5_O_2_
**S8**	4-F	85.48	198–199	448.21	C_25_H_26_FN_5_O_2_
**S9**	2-CH_3_	43.56	222–223	444.24	C_26_H_29_N_5_O_2_
**S10**	3-CH_3_	86.98	106–107	444.24	C_26_H_29_N_5_O_2_
**S11**	3-OCH_3_	95.26	168–169	459.24	C_26_H_29_N_5_O_3_
**S12**	4-OCH_3_	78.13	178–179	459.24	C_26_H_29_N_5_O_3_
**S13**	4-N(CH_3_)_2_	72.18	108–109	472.26	C_27_H_32_N_6_O_2_
**S14**	4-NO_2_	59.51	199–200	474.21	C_25_H_26_N_6_O_4_
**S15**	4-C_2_H_5_	78.62	225–226	458.26	C_27_H_31_N_5_O_2_
**S16**	2-CN	66.85	189–190	455.22	C_26_H_26_N_6_O_2_
**S17**	3-CN	86.49	128–129	455.22	C_26_H_26_N_6_O_2_
**S18**	4-CN	69.47	129–130	455.22	C_26_H_26_N_6_O_2_
**S19**	2-OCH_3_	92.37	206–207	459.24	C_26_H_29_N_5_O_3_
**S20**	3,5-diOCH_3_	97.37	208–209	490.25	C_27_H_31_N_5_O_4_
**S21**	2,4,6-triOCH_3_	54.81	160–161	520.26	C_28_H_33_N_5_O_5_
**S22**	2,3-diOCH_3_	76.95	212–213	490.25	C_27_H_31_N_5_O_4_
**S23**	3,4,5-triOCH_3_	86.16	146–147	520.26	C_28_H_33_N_5_O_5_
**S24**	2,4-diOCH_3_	61.26	174–175	490.25	C_27_H_31_N_5_O_4_

**Table 2:** The inhibition of MAO-A and MAO-B by the S series ^a^.

Compound	Residual Activity at 10 µM (%)		IC_50_ (µM)		SI ^b^
	MAO-A	MAO-B	MAO-A	MAO-B	
**S1**	63.41 ± 2.24	36.47 ± 8.32	16.469 ± 0.047	7.046 ± 0.086	2.34
**S2**	54.76 ± 3.10	56.47 ± 4.99	10.949 ± 1.194	10.744 ± 0.565	1.02
**S3**	34.35 ± 1.93	50.59 ± 0.01	4.128 ± 1.124	4.753 ± 0.155	0.87
**S4**	56.91 ± 9.78	33.53 ± 2.50	26.966 ± 0.797	6.171 ± 0.155	4.37
**S5**	25.58 ± 3.85	−24.57 ± 8.51	3.857 ± 0.770	0.203 ± 0.004	19.04
**S6**	42.22 ± 3.22	26.71 ± 3.83	7.136 ± 1.066	4.955 ± 0.353	1.44
**S7**	12.81 ± 7.24	1.03 ± 4.63	4.739 ± 0.624	1.711 ± 0.009	2.77
**S8**	22.36 ± 3.07	31.85 ± 2.62	7.994 ± 1.281	5.371 ± 0.123	1.49
**S9**	23.19 ± 1.20	32.89 ± 5.19	4.508 ± 0.589	7.237 ± 0.994	0.62
**S10**	16.14 ± 4.26	9.31 ± 2.04	4.526 ± 0.424	3.549 ± 0.001	1.28
**S11**	16.61 ± 9.23	14.52 ± 1.14	5.064 ± 0.006	1.709 ± 0.437	2.96
**S12**	7.71 ± 8.25	38.46 ± 0.36	4.642 ± 0.001	6.463 ± 0.035	0.72
**S13**	36.08 ± 1.01	37.56 ± 2.50	5.634 ± 1.182	6.480 ± 0.559	0.87
**S14**	67.08 ± 5.19	39.77 ± 4.14	24.170 ± 0.283	3.624 ± 0.540	6.67
**S15**	30.12 ± 8.10	54.44 ± 4.05	3.691 ± 0.655	5.336 ± 0.667	0.69
**S16**	33.16 ± 9.25	−12.90 ± 1.14	6.149 ± 0.305	0.979 ± 0.206	6.28
**S17**	9.98 ± 0.57	0.00 ± 0.01	6.283 ± 0.306	3.099 ± 0.088	2.03
**S18**	35.94 ± 2.54	48.37 ± 0.08	7.192 ± 0.201	3.513 ± 0.811	2.05
**S19**	7.83 ± 2.05	20.76 ± 1.76	4.662 ± 0.307	1.000 ± 0.326	4.66
**S20**	68.02 ± 4.21	11.71 ± 2.41	18.999 ± 2.714	3.631 ± 0.142	5.23
**S21**	32.79 ± 2.18	48.45 ± 6.43	7.471 ± 0.344	6.123 ± 0.662	1.22
**S22**	−8.50 ± 2.12	39.00 ± 2.83	5.938 ± 0.264	7.871 ± 0.208	0.75
**S23**	78.79 ± 0.30	53.11 ± 2.67	28.373 ± 2.953	10.949 ± 0.405	2.59
**S24**	20.70 ± 1.34	54.66 ± 3.76	4.128 ± 0.107	10.660 ± 2.476	0.39
Toloxatone	11.72 ± 0.39	81.89 ± 2.68	1.245 ± 0.233	>40	>32.13
Clorgyline	−0.30 ± 0.43	25.84 ± 0.23	0.013 ± 0.008	1.853 ± 0.112	142.54
Safinamide	69.04 ± 1.36	−15.61 ± 0.86	>40	0.021 ± 0.001	>1904
Pargyline	19.88 ± 6.82	−11.08 ± 1.53	2.403 ± 0.358	0.14 ± 0.01	17.16

^a^ Results are means ± standard errors of duplicate or triplicate experiments. ^b^ Selectivity index (SI) values are expressed as IC_50_ of MAO-B versus IC_50_ of MAO-A.

**Table 3:** Blood–brain barrier assay of key compounds using PAMPA method.

Compound	Experimental Pe (×10^−6^ cm·s^−1^)	Prediction
**S5**	5.75 ± 0.13	CNS+
**S16**	3.78 ± 0.20	CNS+
Selegiline	5.69 ± 0.04	CNS+

Pe (10^−6^ cm·s^−1^) > 4.00: CNS+ (high permeation); Pe (10^−6^ cm·s^−1^) < 2.00: CNS− (low permeation); Pe (10^−6^ cm·s^−1^) from 2.00 to 4.00: CNS± (BBB permeation uncertain).

## Data Availability

Data are contained within the article and Supplementary Materials.

## Supplementary Materials

The following supporting information can be downloaded at https://www.mdpi.com/article/10.3390/molecules29133097/s1, Figures S1–S16: Structures of sixteen compounds comprising ^1^H-NMR spectrum, ^13^C-NMR spectrum, and ESI-MS spectrum.
