# Supplementary material for: Inhibition of Monoamine Oxidases by Pyridazinobenzylpiperidine Derivatives
Source: Molecules. 2024 Jun 28;29(13):3097. doi: 10.3390/molecules29133097 (PMC11243598; doi:10.3390/molecules29133097)

Supplementary Information

Inhibitions of monoamine oxidases by pyridazinobenzylpiperidine derivatives

Jong Min Oh[a#], Yaren Nur ZENNİ[b#], Zeynep Özdemir[b*], Sunil Kumar[c], Semanur KILIÇ[b], Mevlüt AKDAĞ[d], Azime Berna ÖZÇELİK[e], Hoon Kim[a*] , Bijo Mathew[c*]

[a] Department of Pharmacy, and Research Institute of Life Pharmaceutical Sciences, Sunchon National University, Suncheon 57922, Republic of Korea.

[b]Department of Pharmaceutical □hemistry, Faculty of Pharmacy, Inonu University, Malatya, 44210, Türkiye.

[c] Department of Pharmaceutical □hemistry, Amrita School of Pharmacy, Amrita Vishwa Vidyapeetham, AIMS Health Sciences □ampus, Kochi 682 041, India.

[d]Department of Pharmaceutical □hemistry, Faculty of Pharmacy, Afyonkarahisar Health Sciences University, Afyonkarahisar, 03030, Türkiye.

[e]Department of Pharmaceutical □hemistry, Faculty of Pharmacy, □azi University, Ankara, 06100, Türkiye.

# Authors contributed equally.

***Corresponding authors:**

Zeynep Özdemir (Z. Özdemir) (zeynep.bulut@inonu.edu.tr)

Hoon Kim (H. Kim) (hoon@sunchon.ac.kr)

Bijo Mathew (B. Mathew) (bijomathew@aims.amrita.edu) (bijovilaventgu@gmail.com)

Contents: Figures S1 to S16

^{1}H-NMR, ^{13}□-NMR and ESI-MS spectra of compounds **S3-S5, S7, S9, S10, S15-S24**

Figure S1. Compound S3

Data 1. ^{1}H-NMR spectra of compound S3

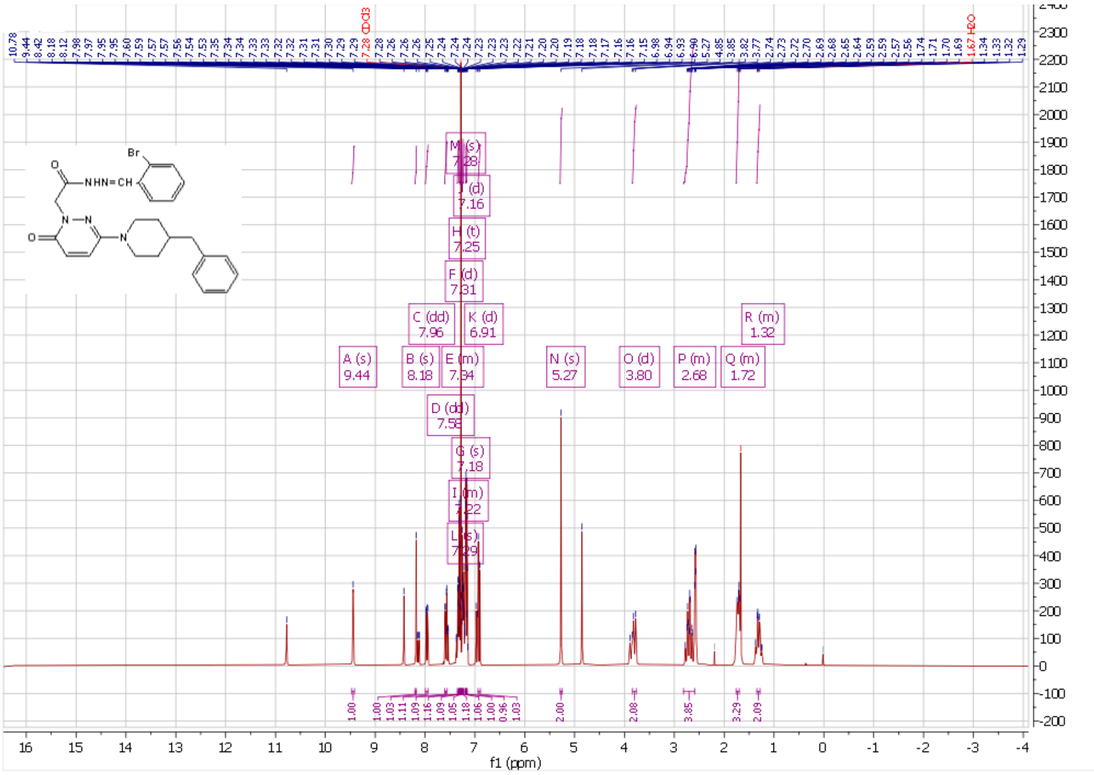

Data 2. ^{13}C-NMR spectra of compound S3

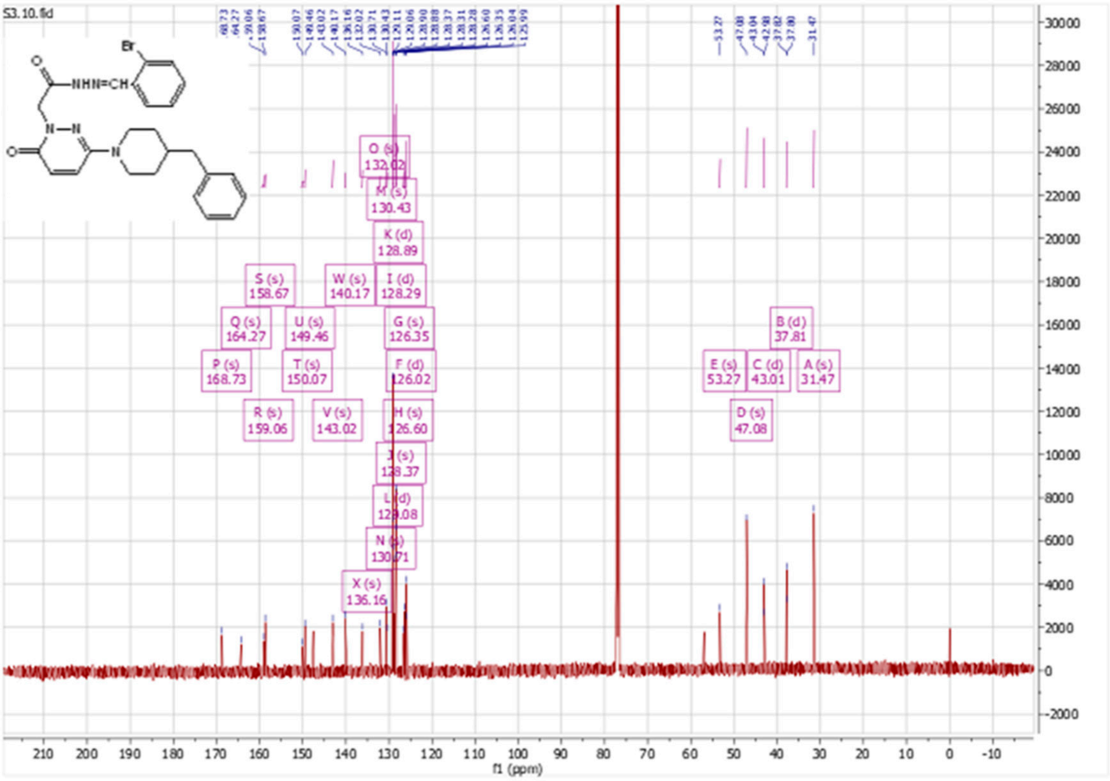

Data 3. HRMS spectra of compound S3

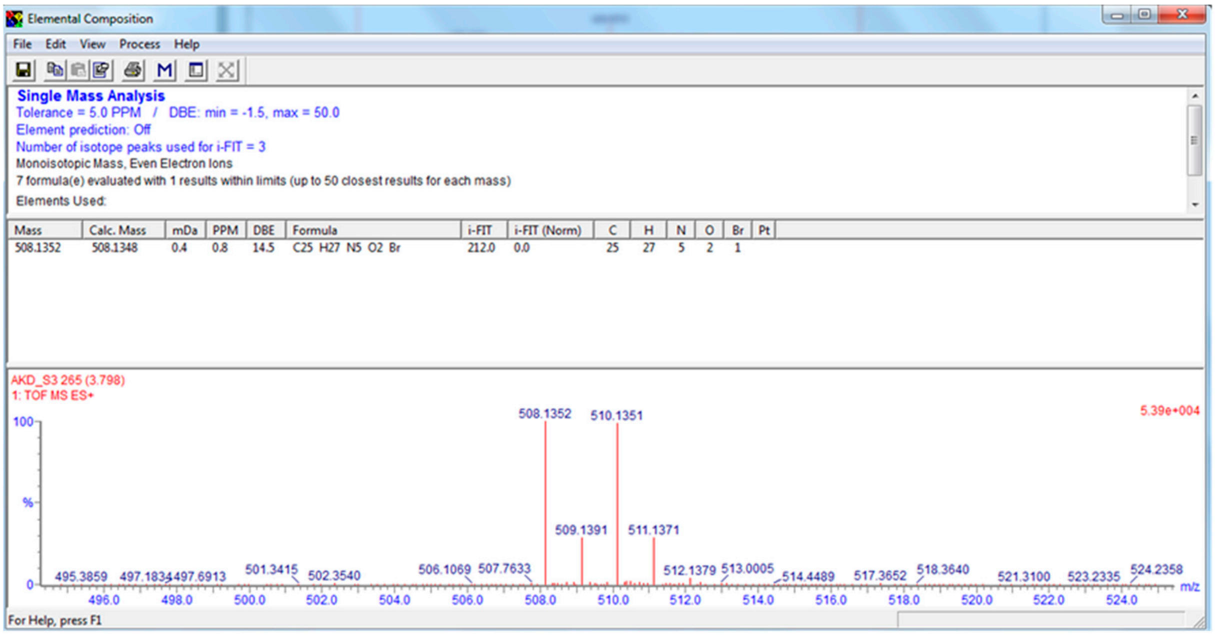

Figure S2. Compound S4

Data 4. ^{1}H-NMR spectra of compound S4

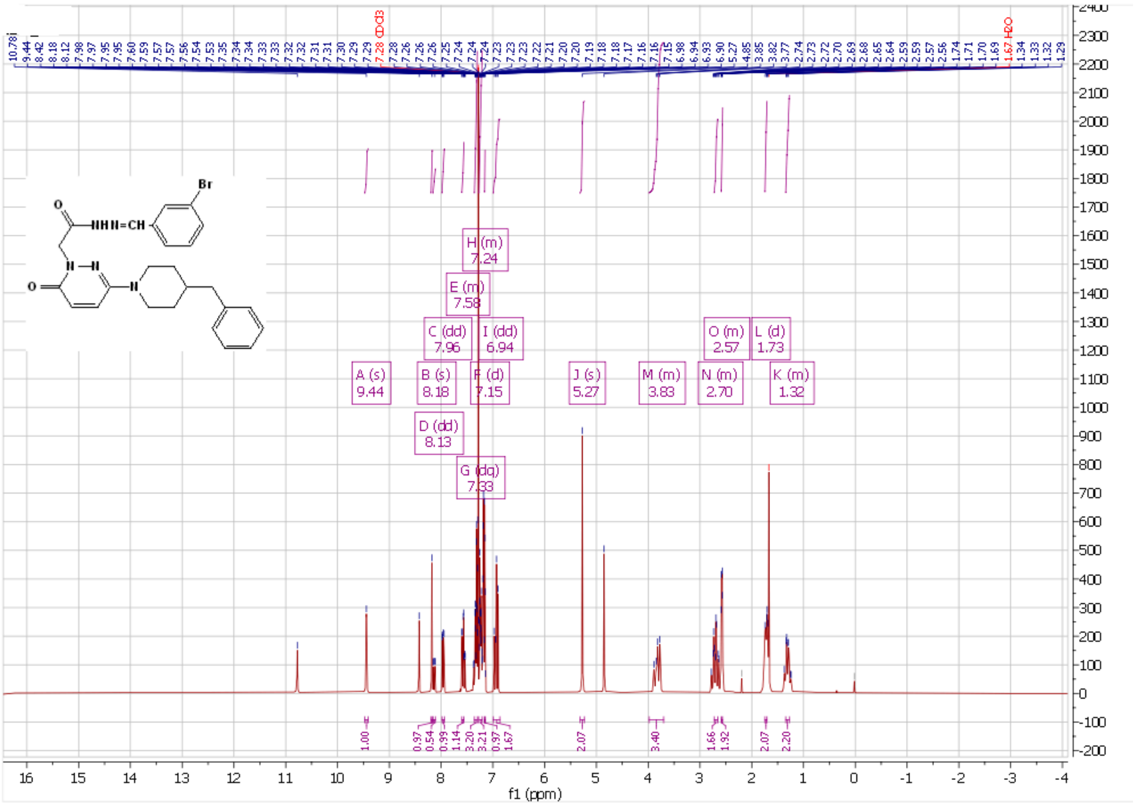

Data 5. ^{13}C-NMR spectra of compound S4

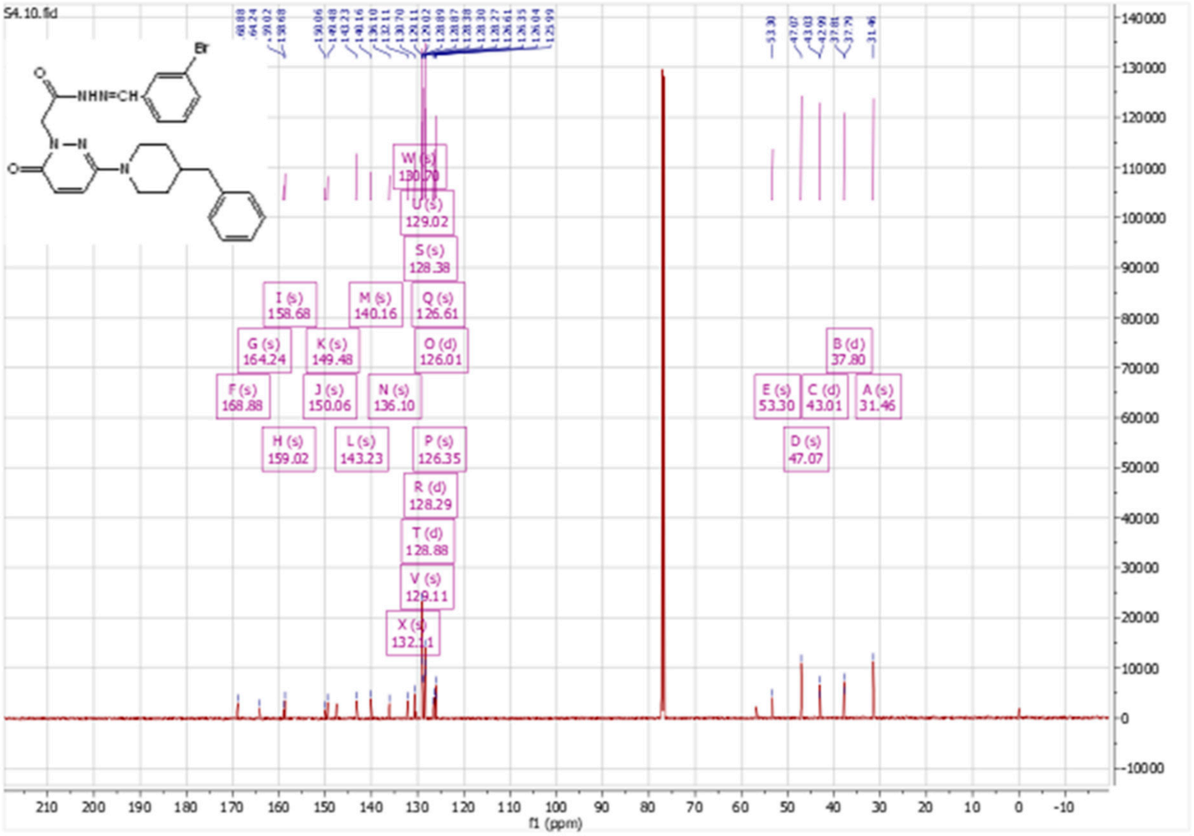

Figure S3. Compound S5

Data 6. ^{1}H-NMR spectra of compound S5

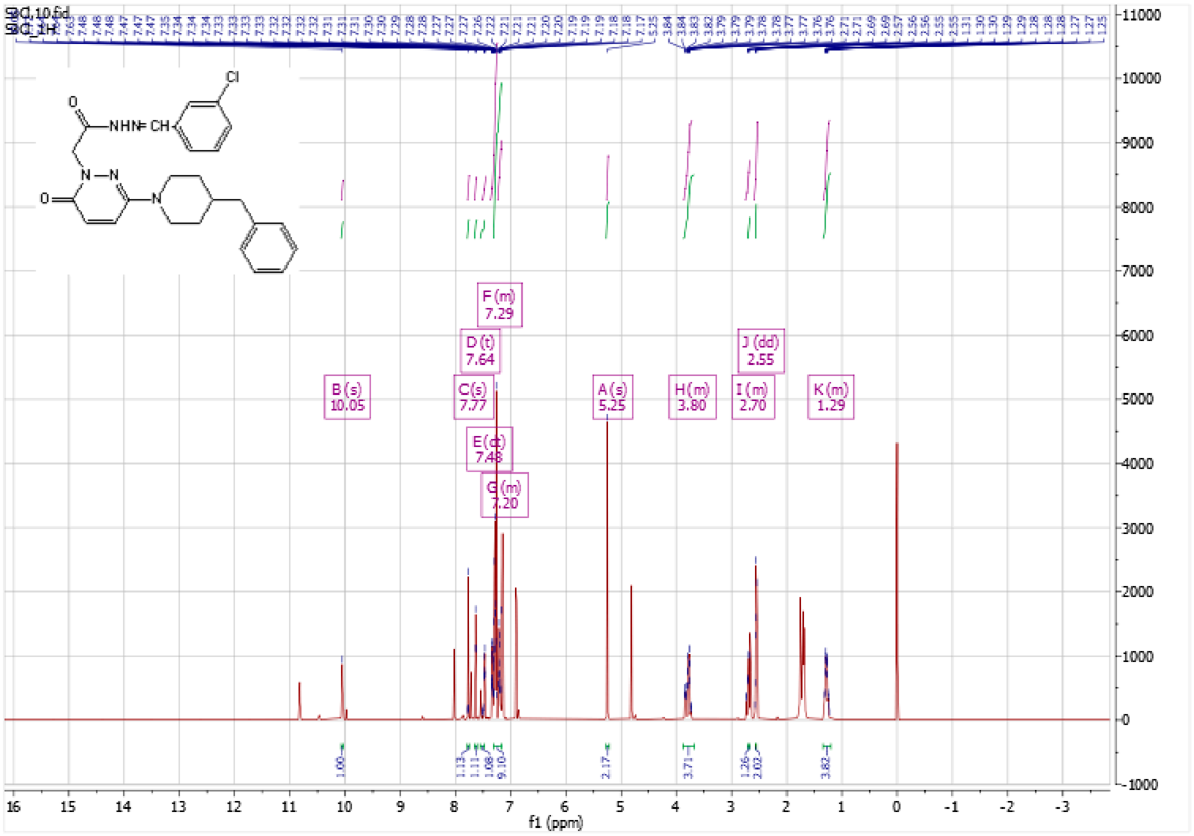

Data 7. ^{13}C-NMR spectra of compound S5

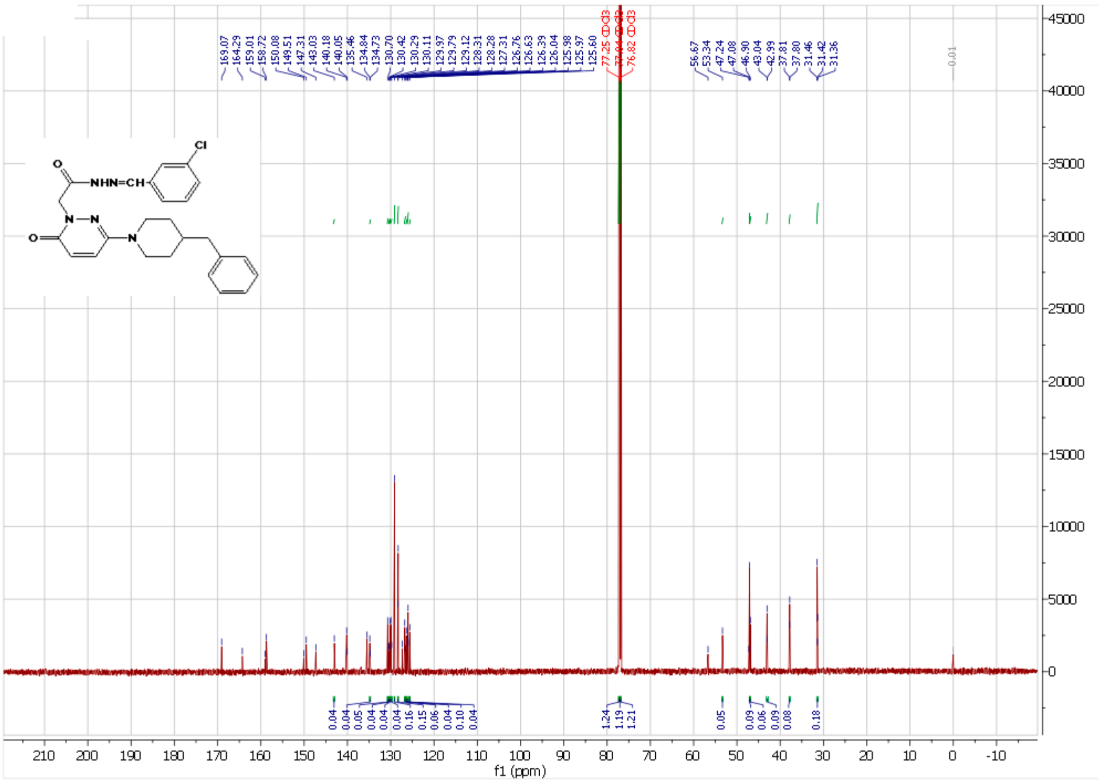

Data 8. HRMS spectra of compound S5

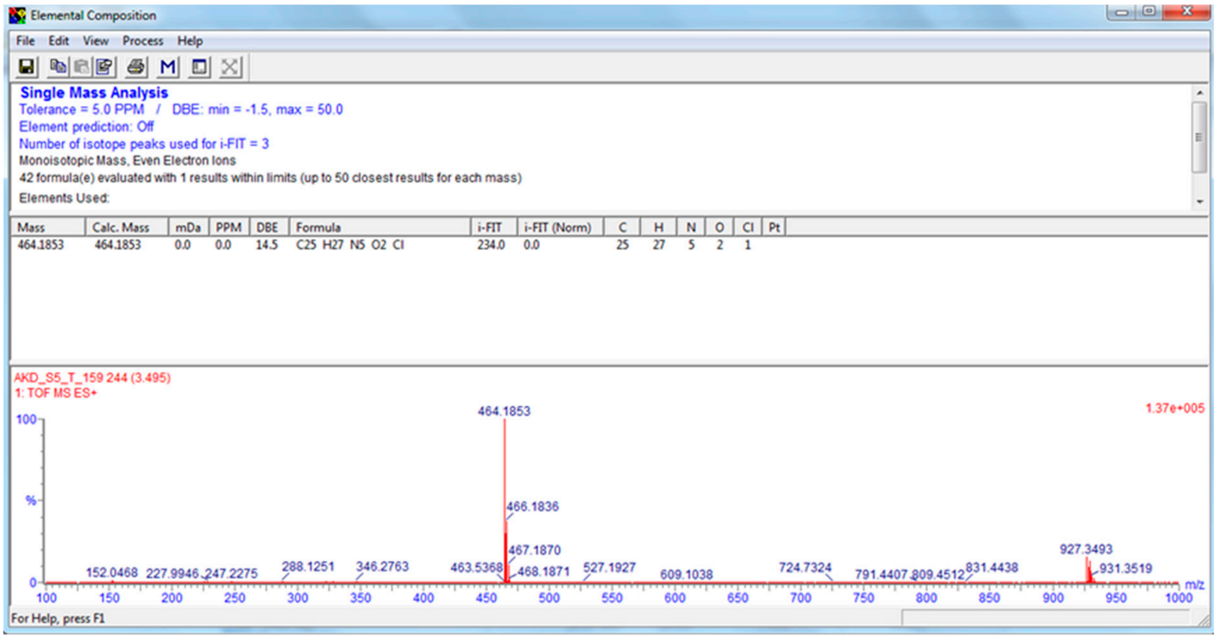

Figure S4. Compound S7

Data 9. ^{1}H-NMR spectra of compound S7

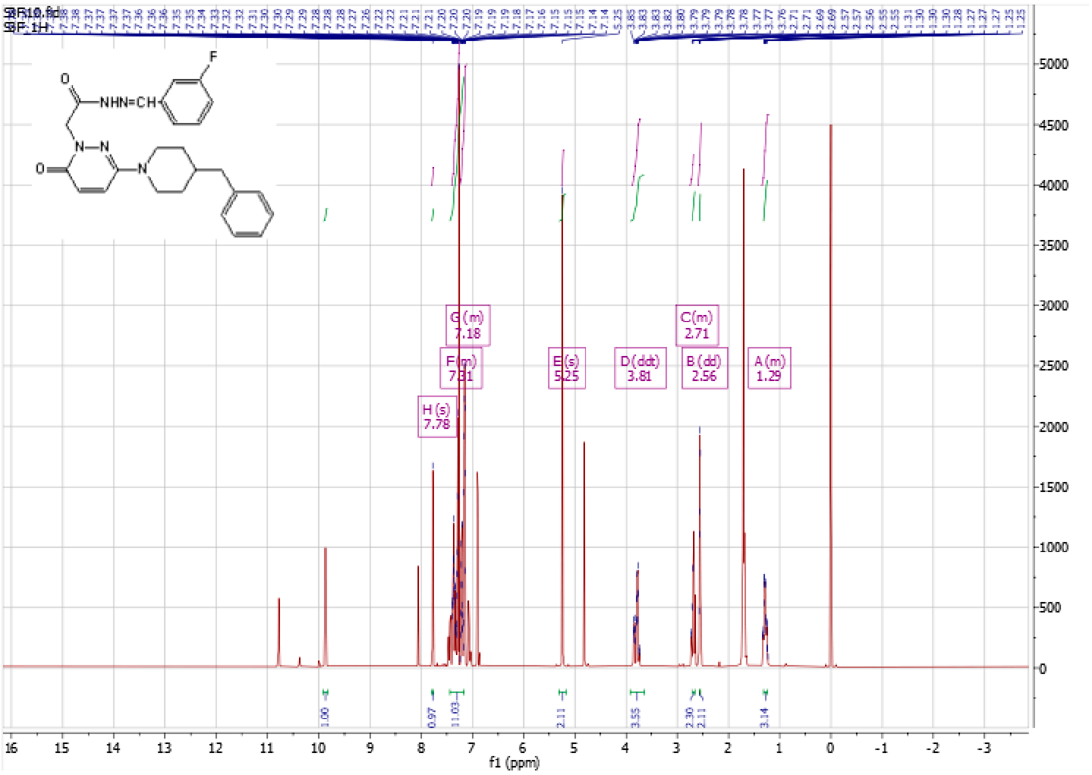

Data 10. ^{13}C-NMR spectra of compound S7

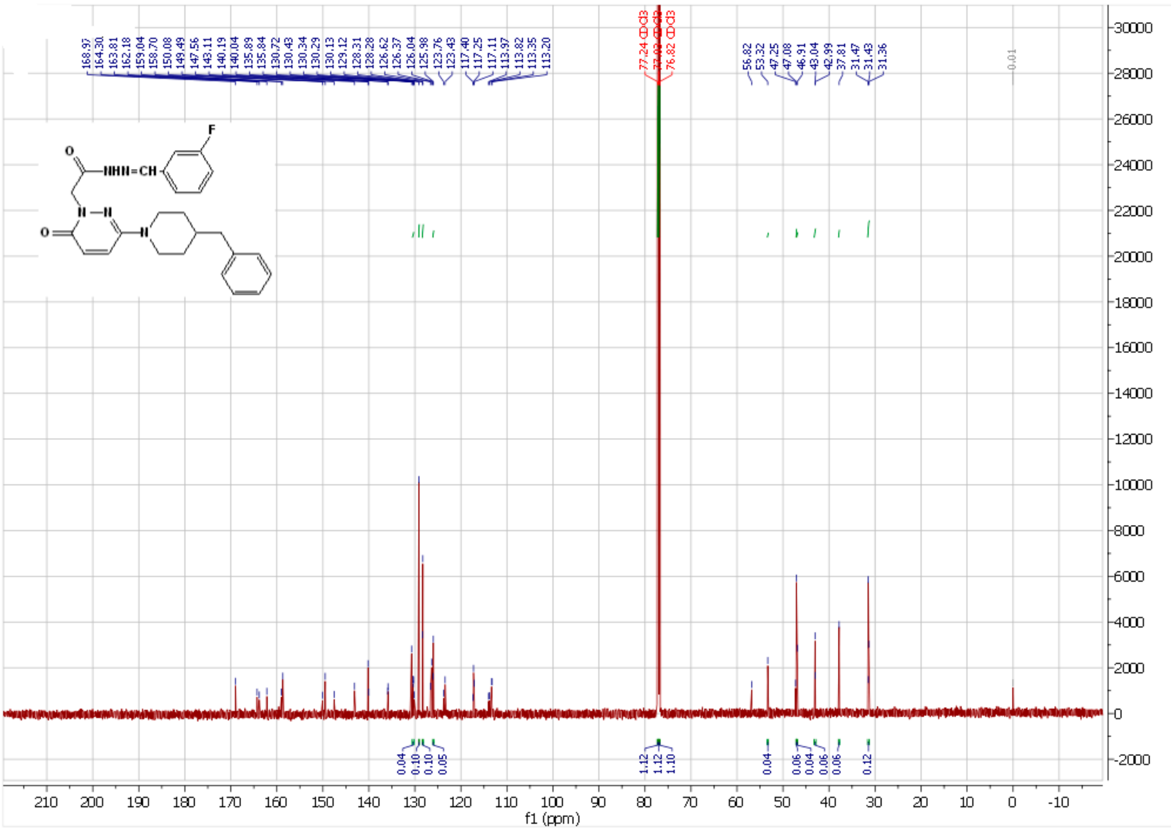

Data 11. HRMS spectra of compound S7

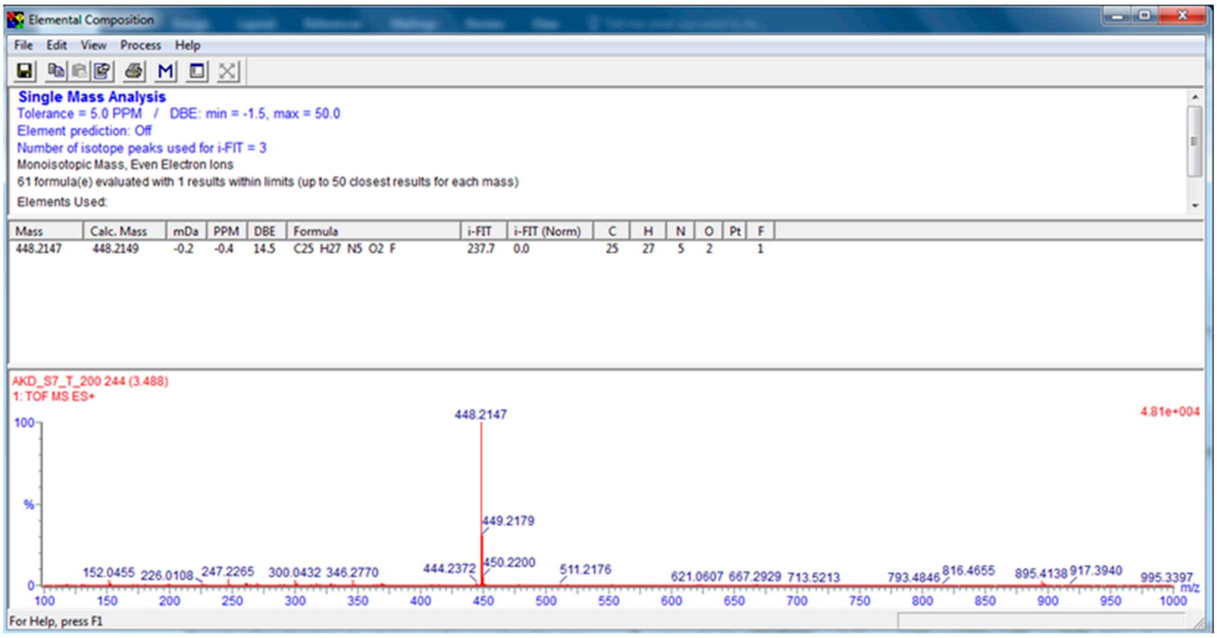

Figure S5. Compound S9

Data 12. ¹H-NMR spectra of compound S9

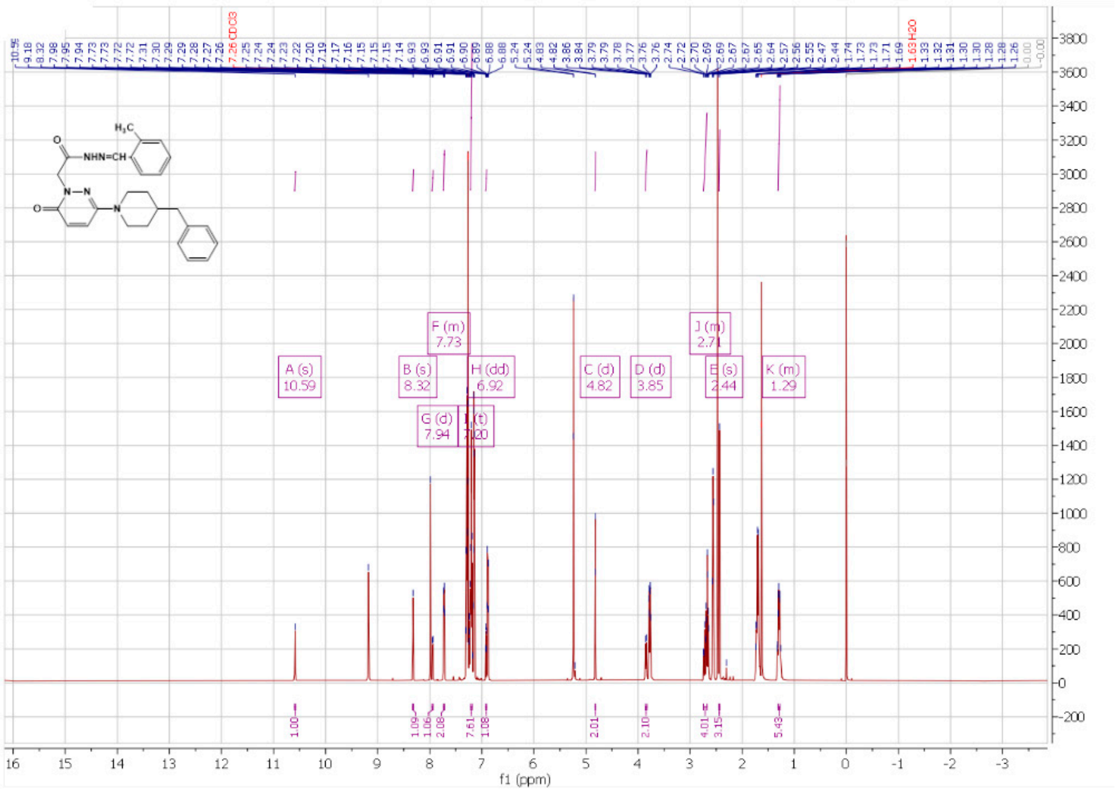

Data 13. ¹³C-NMR spectra of compound S9

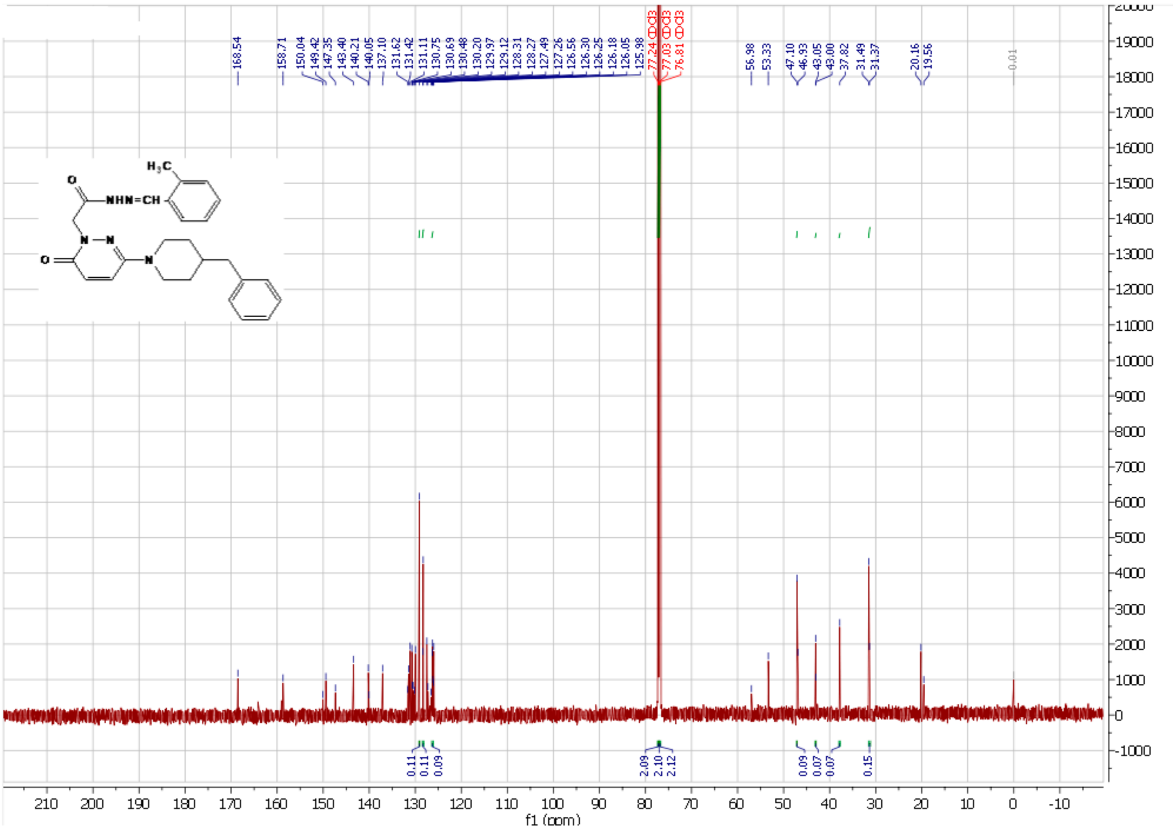

Data 14. HRMS spectra of compound S9

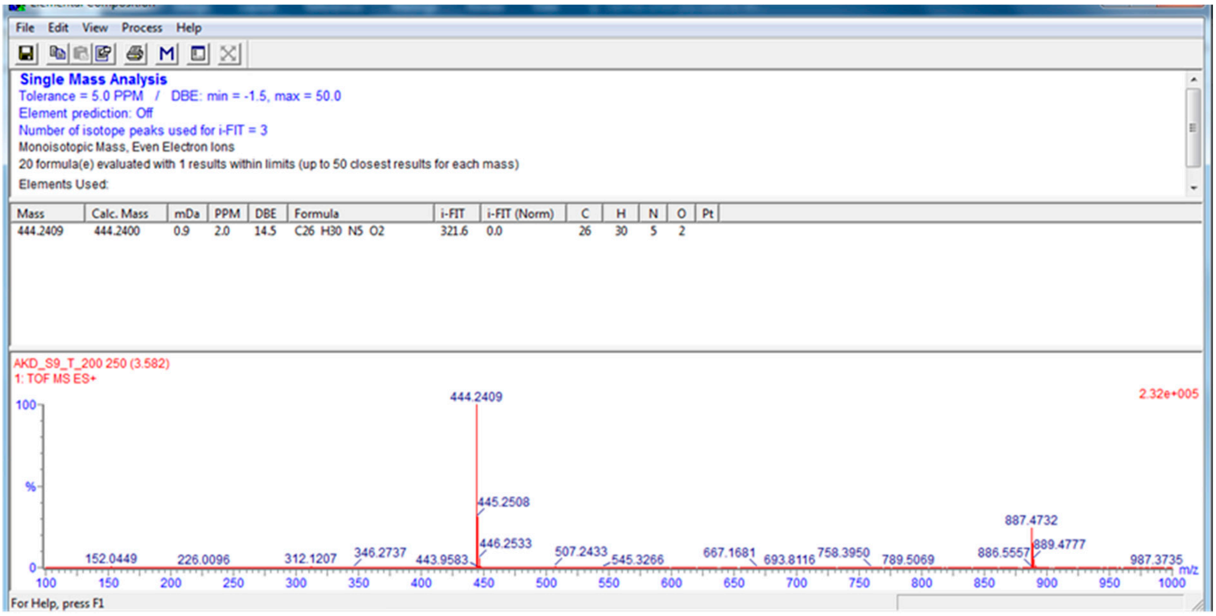

Figure S6. Compound S10

Data 15. ^{1}H-NMR spectra of compound S10

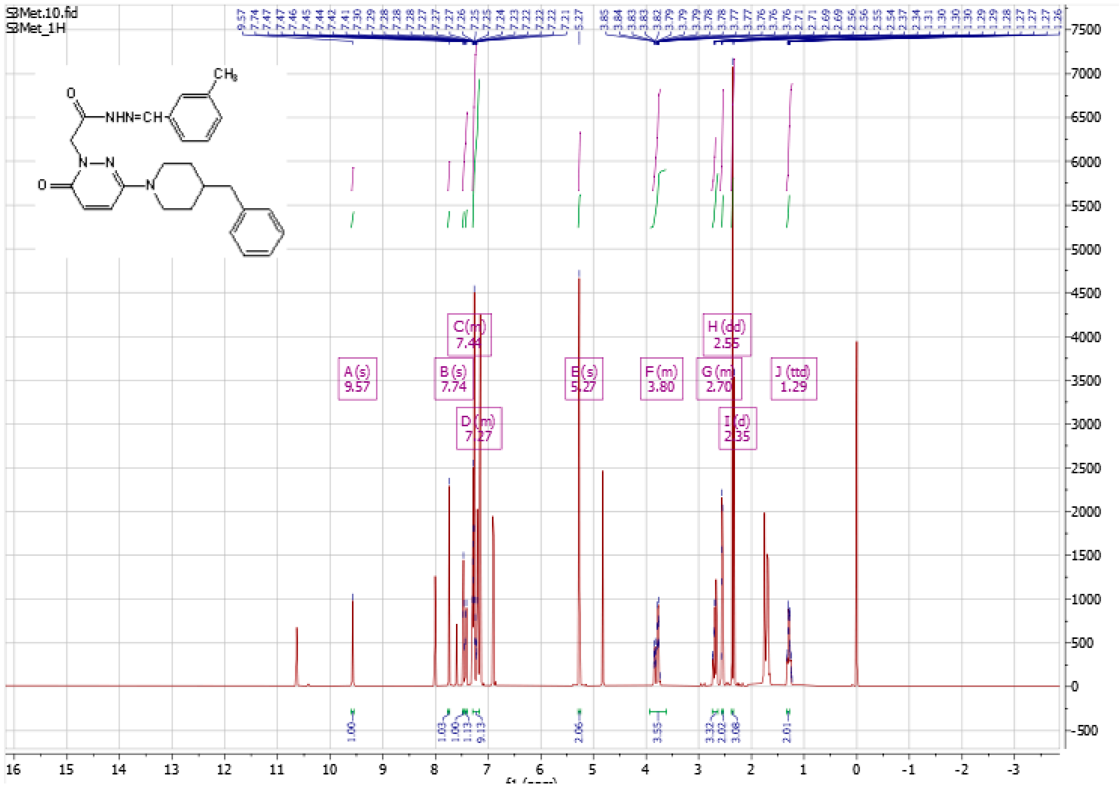

Data 16. ^{13}C-NMR spectra of compound S10

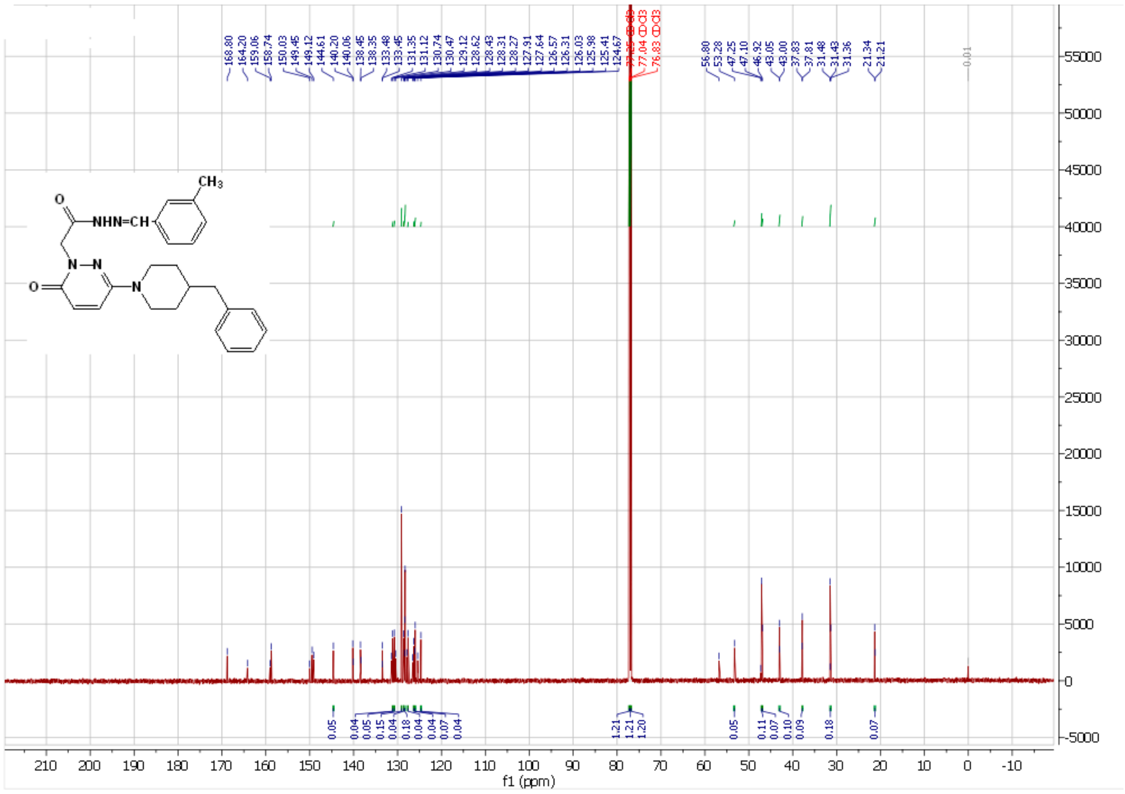

Data 17. HRMS spectra of compound S10

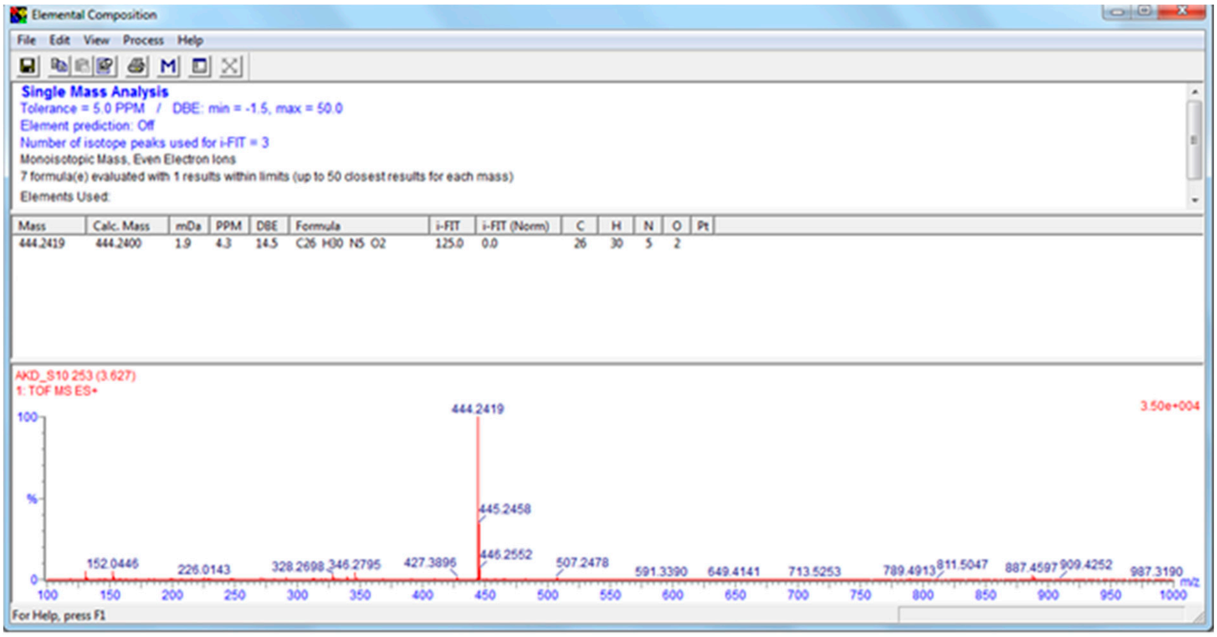

Figure S7. Compound S15

Data 18. ^{1}H-NMR spectra of compound S15

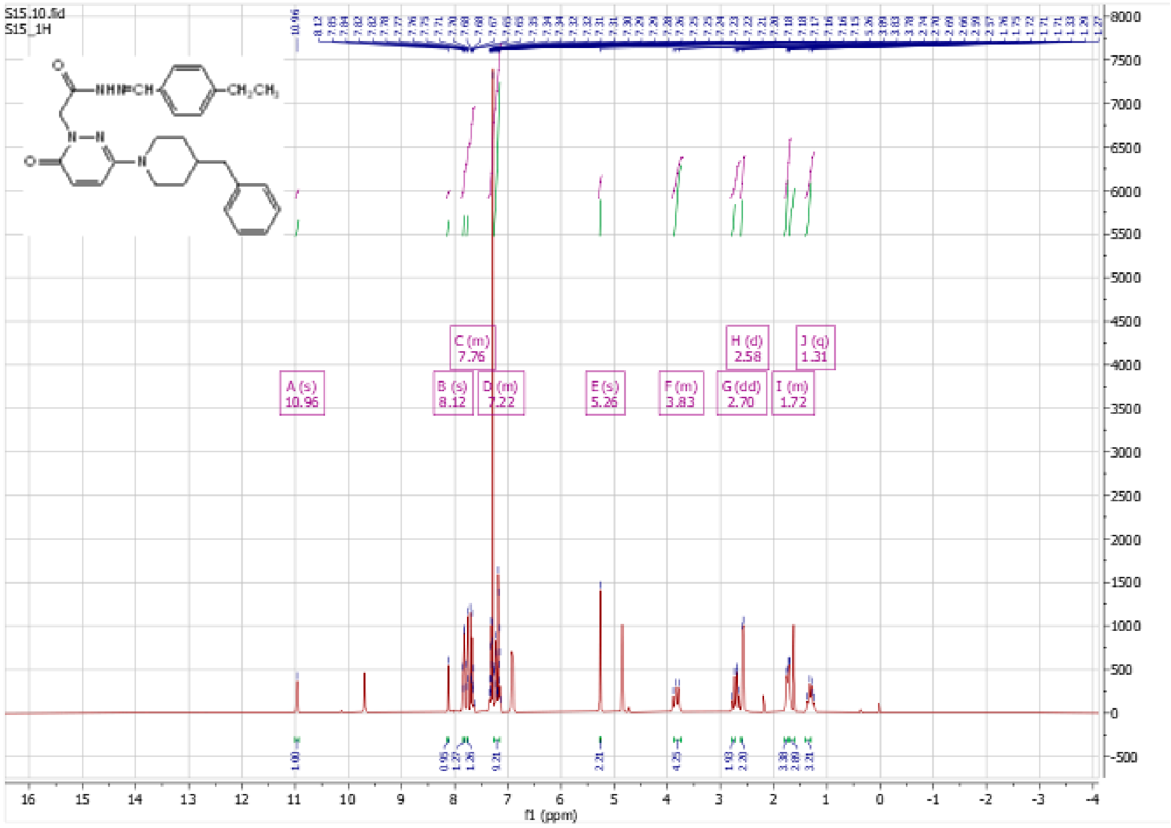

Data 19. ^{13}C-NMR spectra of compound S15

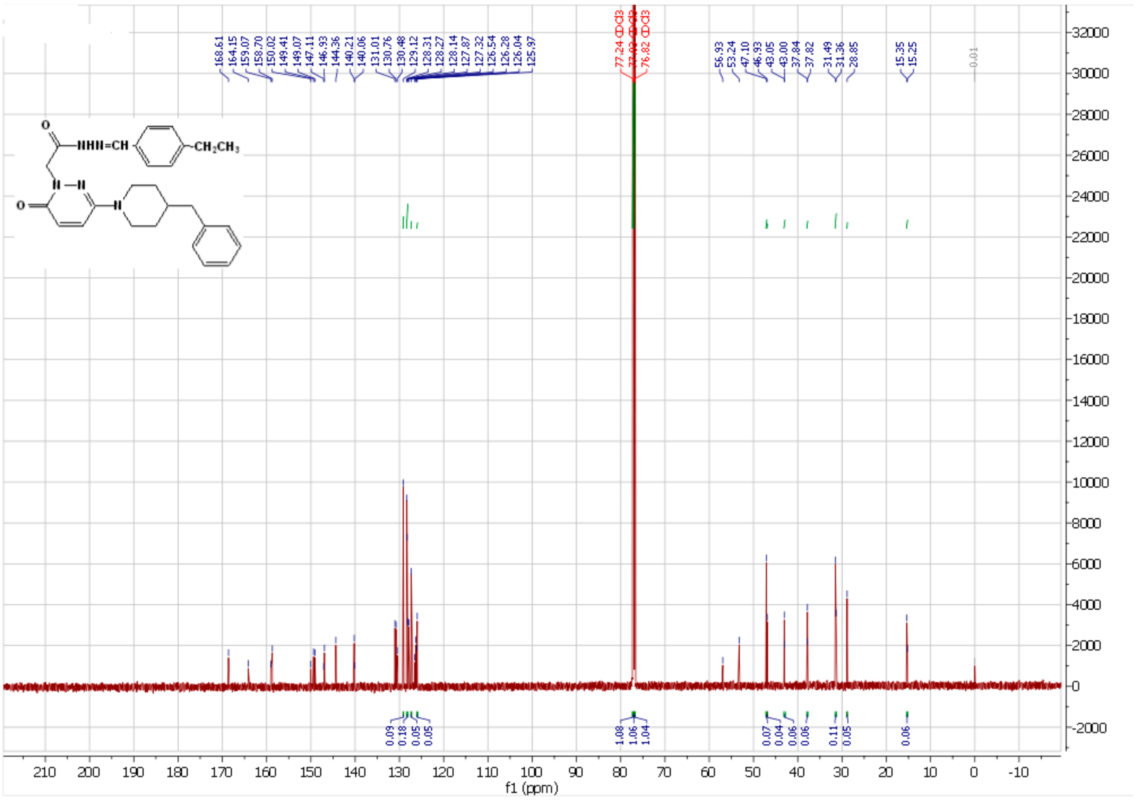

Data 20. HRMS spectra of compound S15

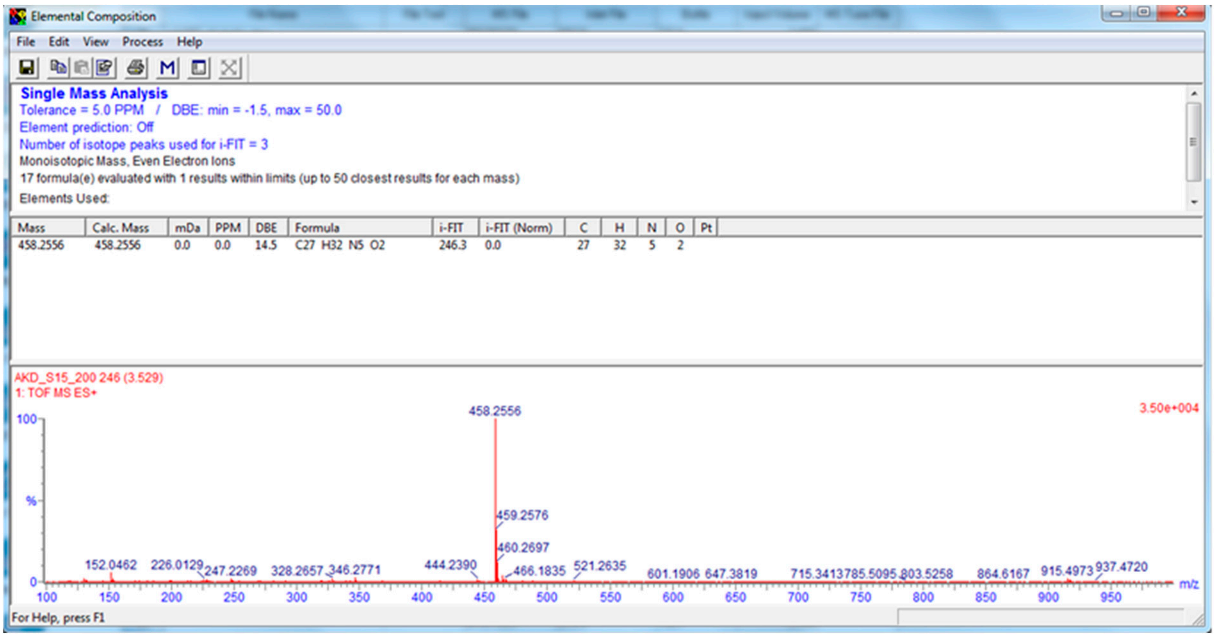

Figure S8. Compound S16

Data 21. ^{1}H-NMR spectra of compound S16

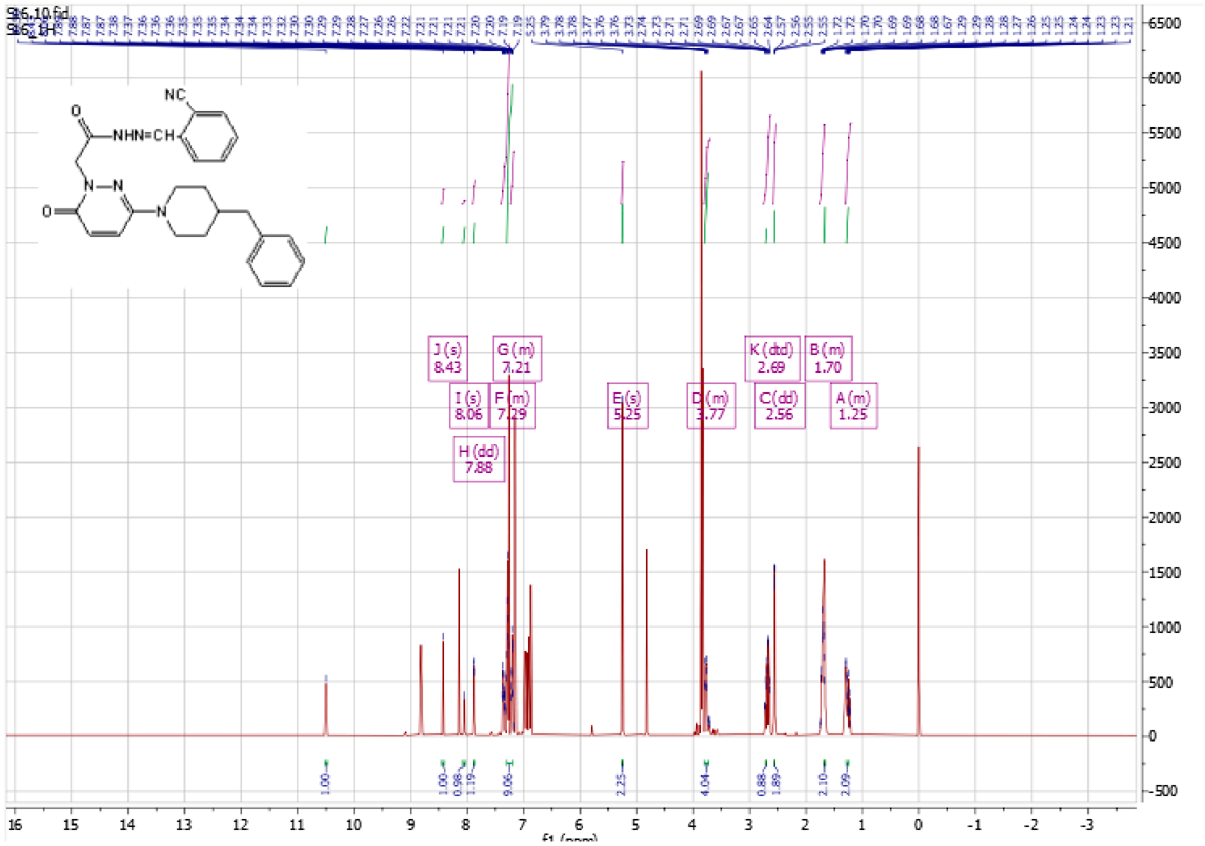

Data 22. ^{13}C-NMR spectra of compound S16

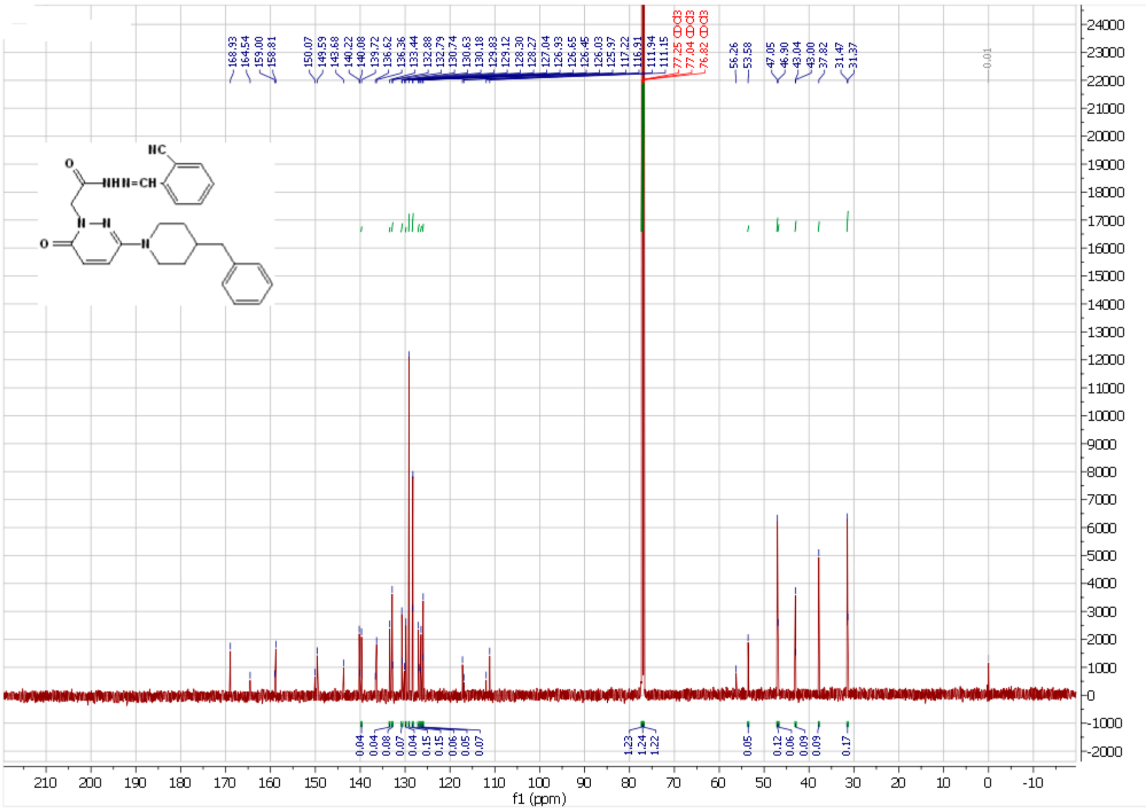

Data 23. HRMS spectra of compound S16

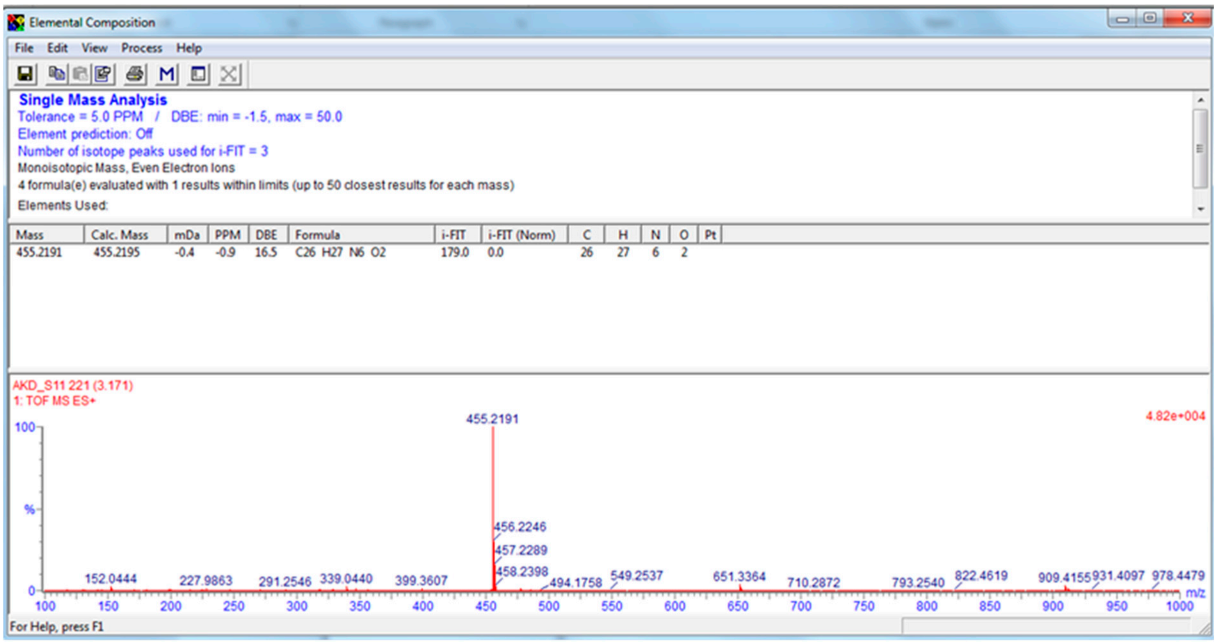

Figure S9. Compound S17

Data 24. ^{1}H-NMR spectra of compound S17

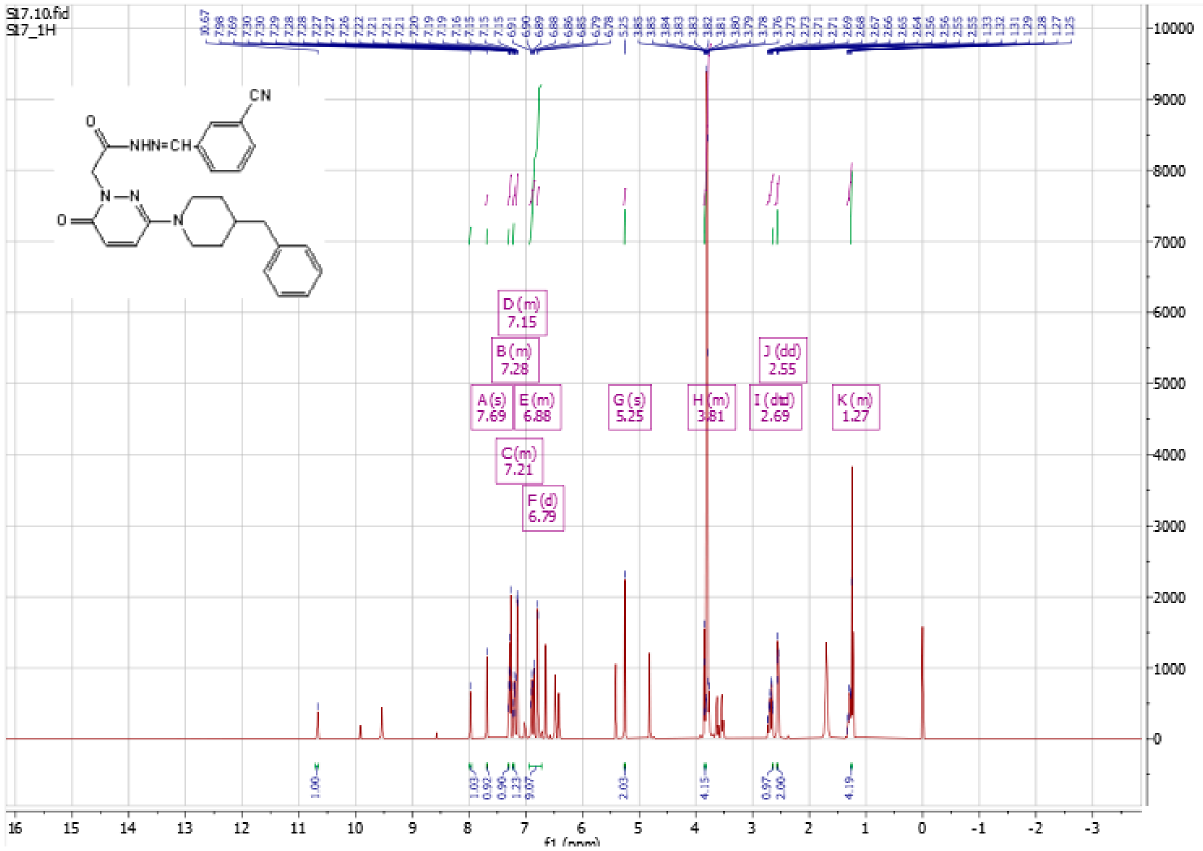

Data 25. ^{13}C-NMR spectra of compound S17

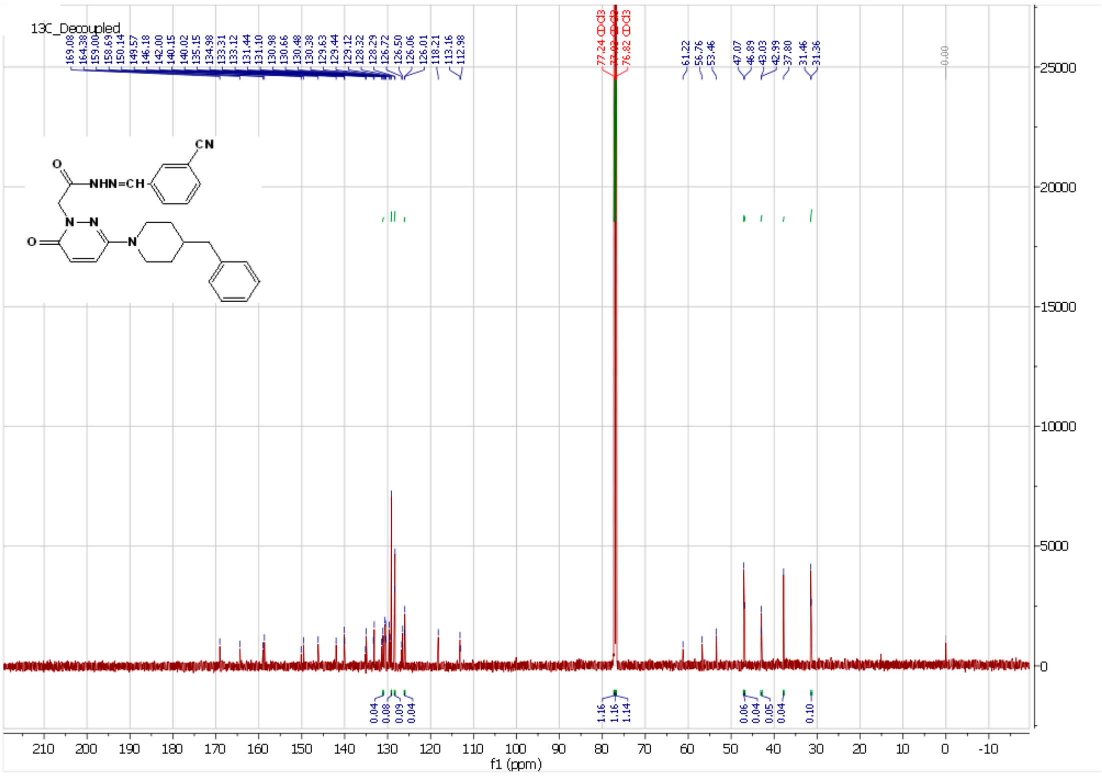

Data 26. HRMS spectra of compound S17

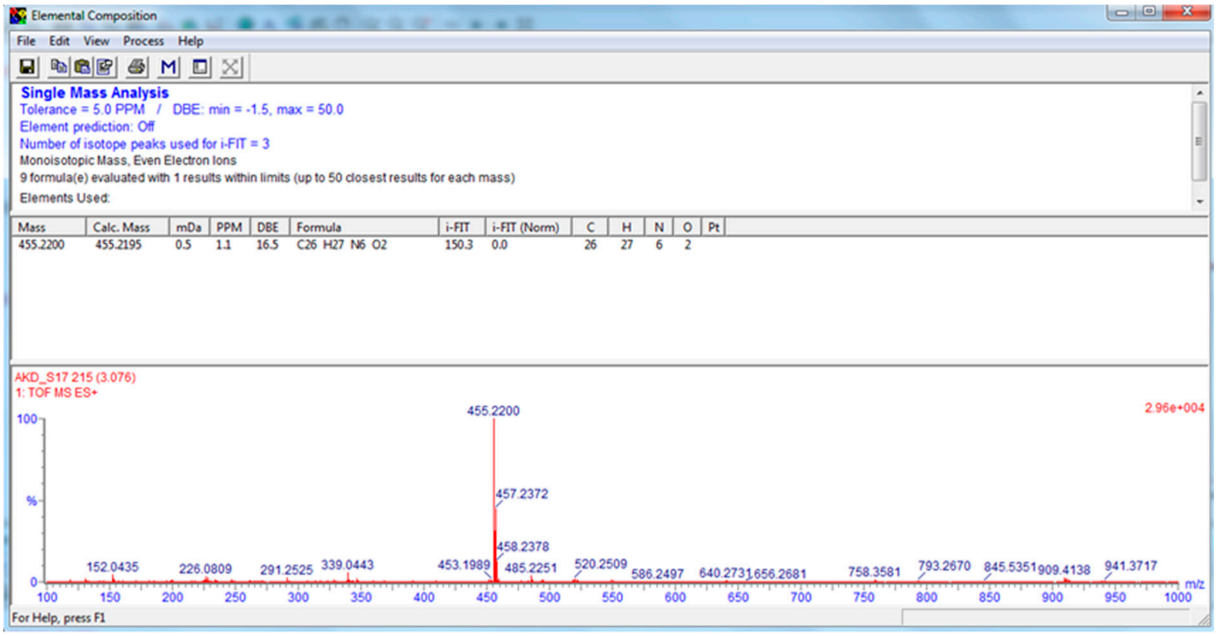

Figure S10. Compound S18

Data 27. ^{1}H-NMR spectra of compound S18

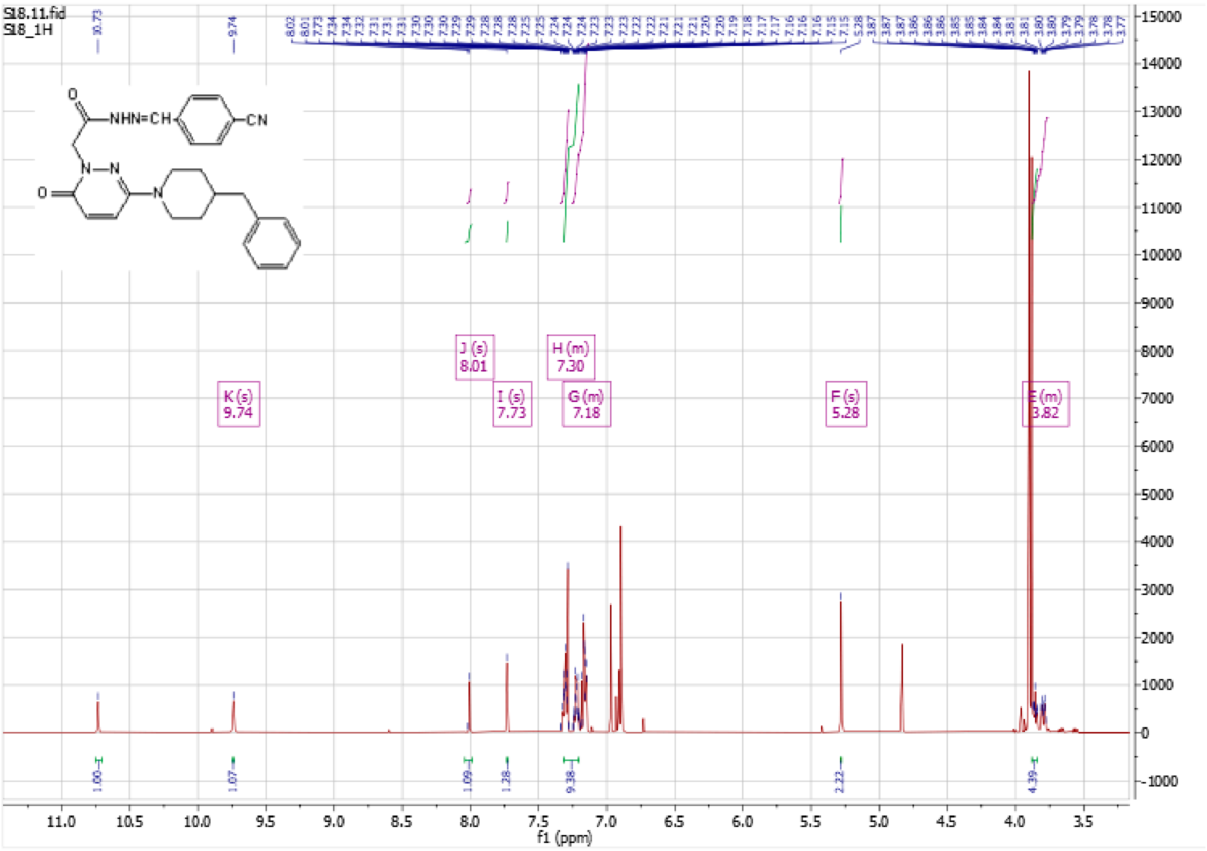

Data 28. ^{13}C-NMR spectra of compound S18

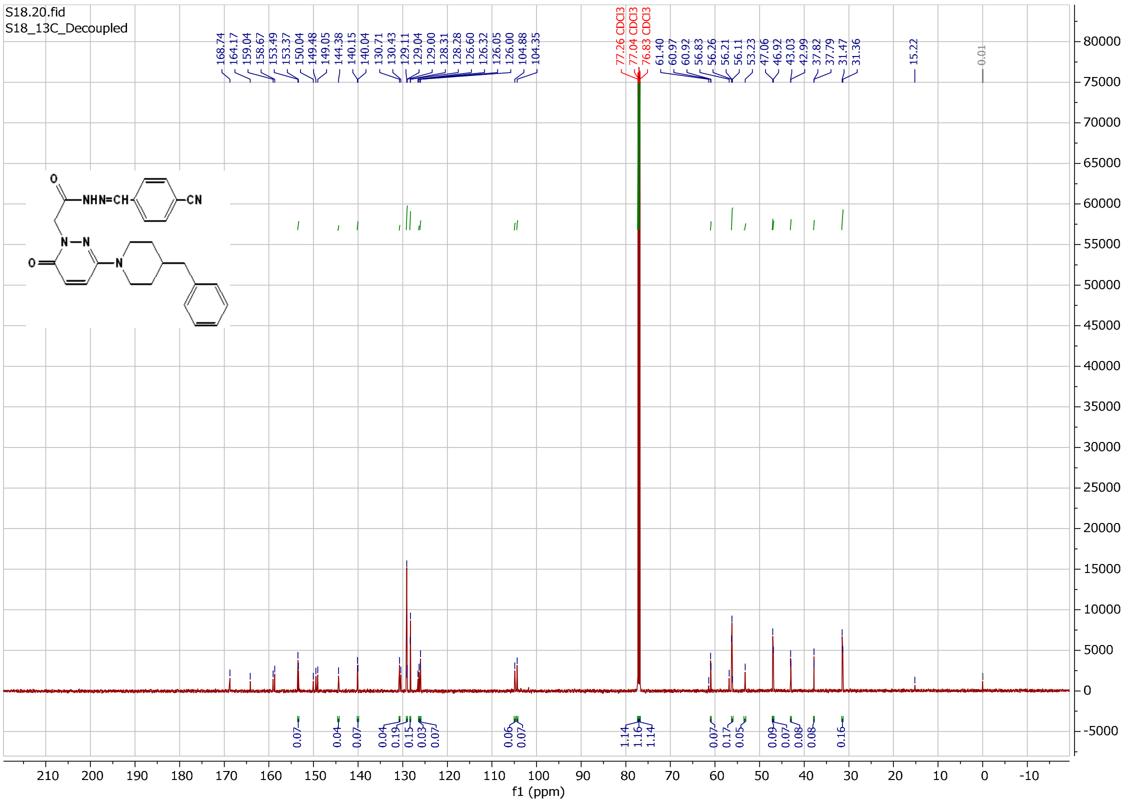

Data 29. HRMS spectra of compound S18

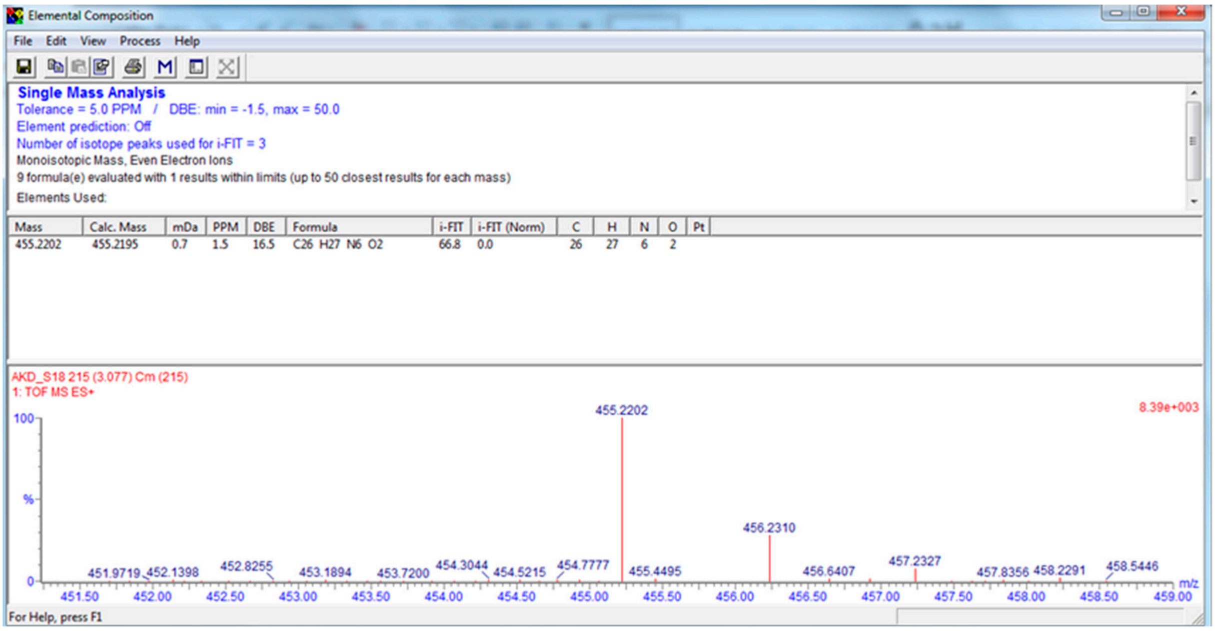

Figure S11. Compound S19

Data 30. ^{1}H-NMR spectra of compound S19

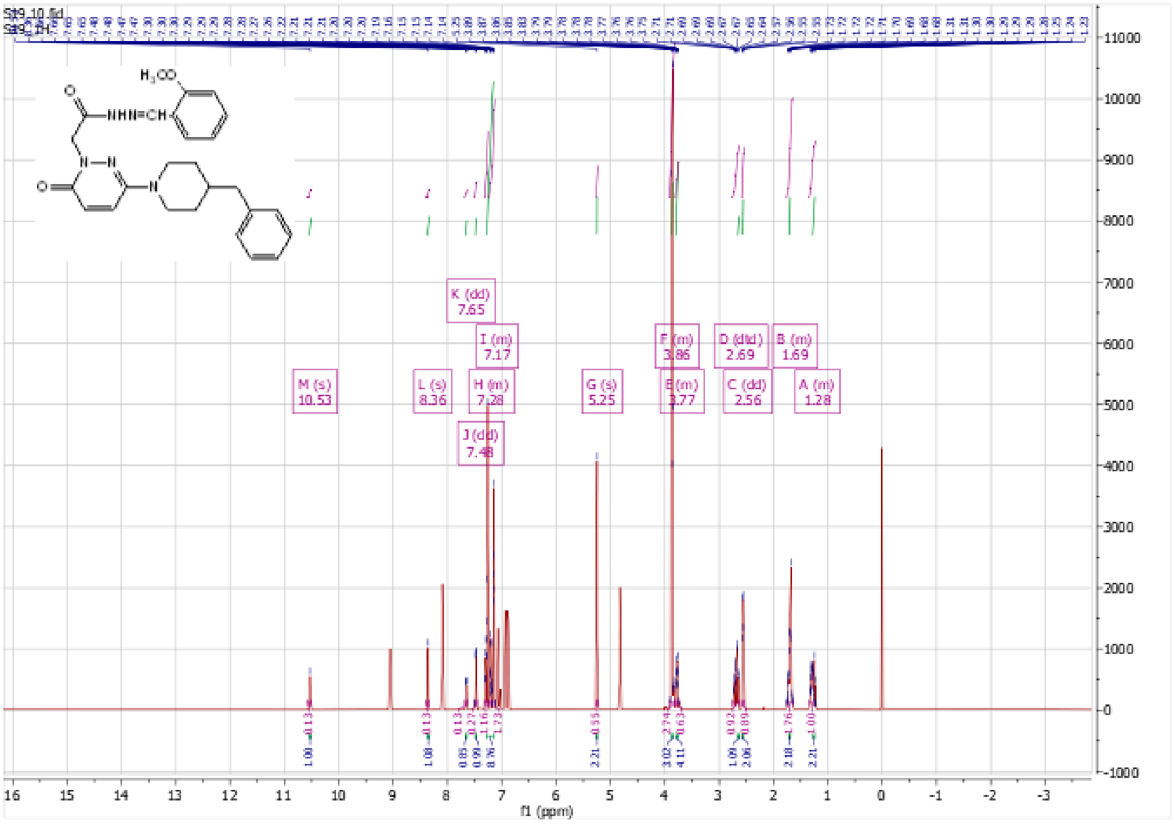

Data 31. ^{13}C-NMR spectra of compound S19

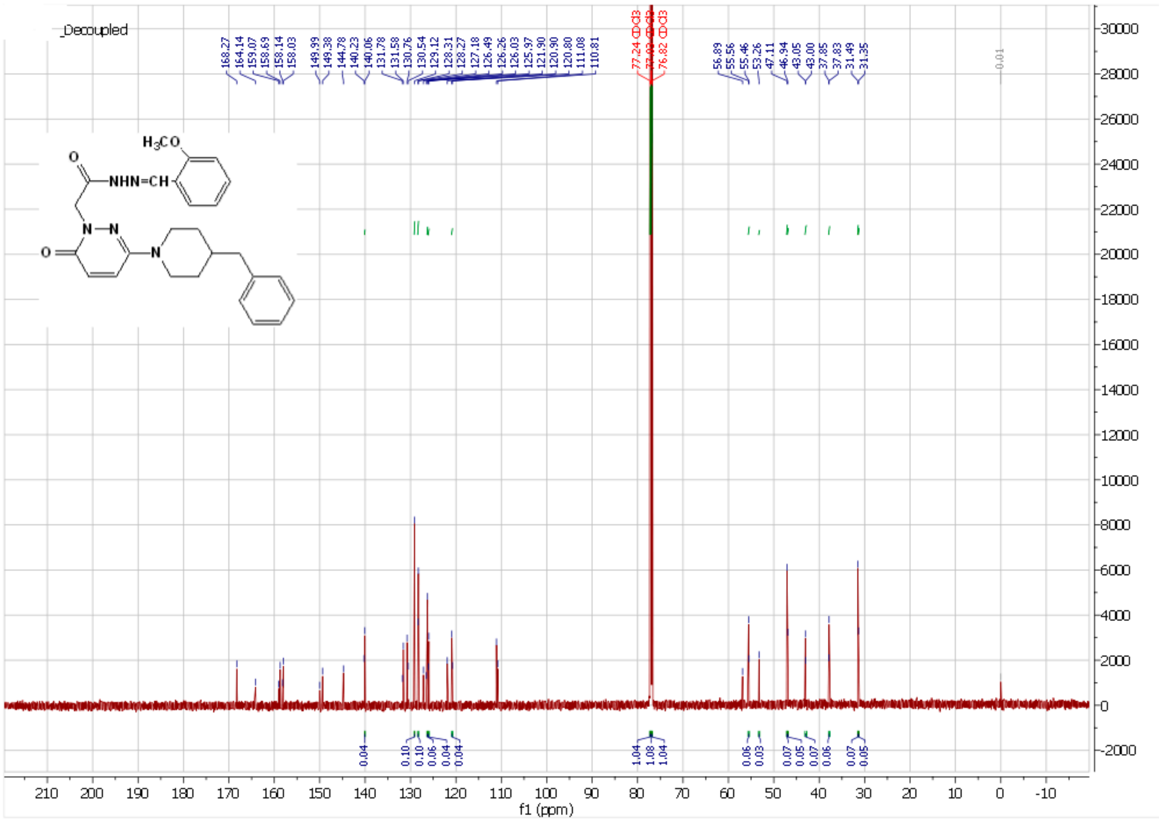

Data 32. HRMS spectra of compound S19

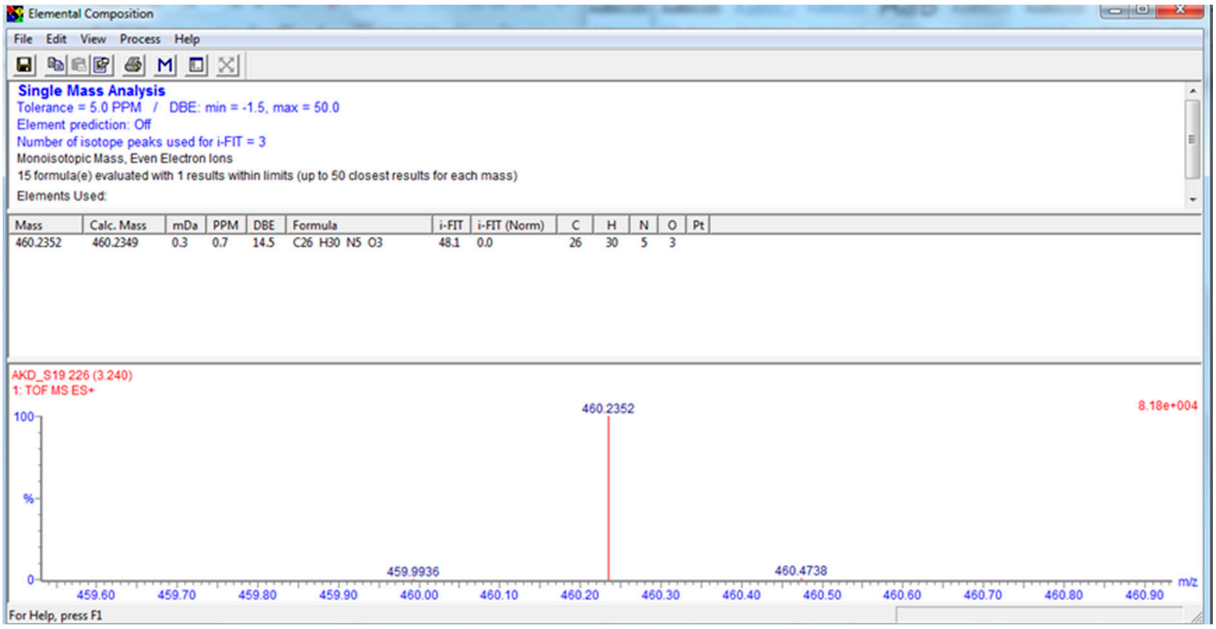

Figure S12. Compound S20

Data 33. ^{1}H-NMR spectra of compound S20

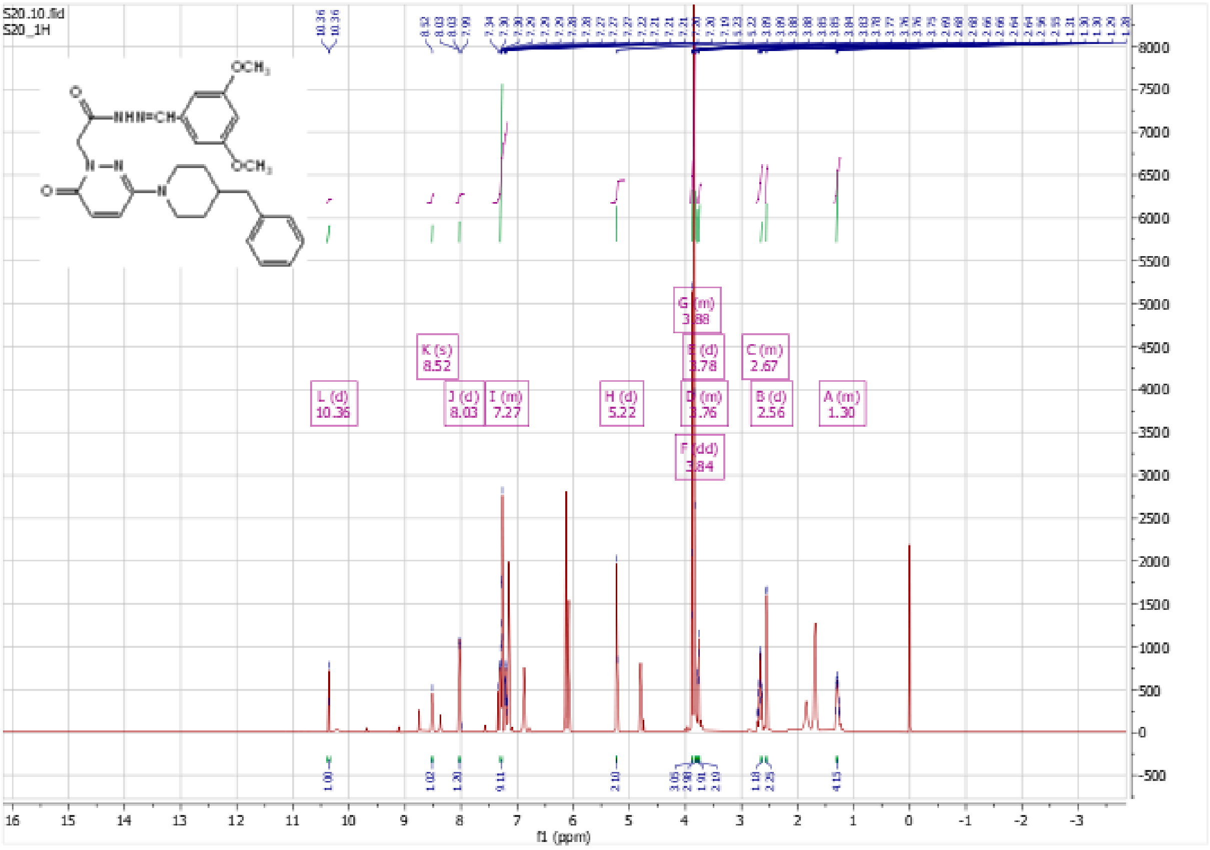

Data 34. ^{13}C-NMR spectra of compound S20

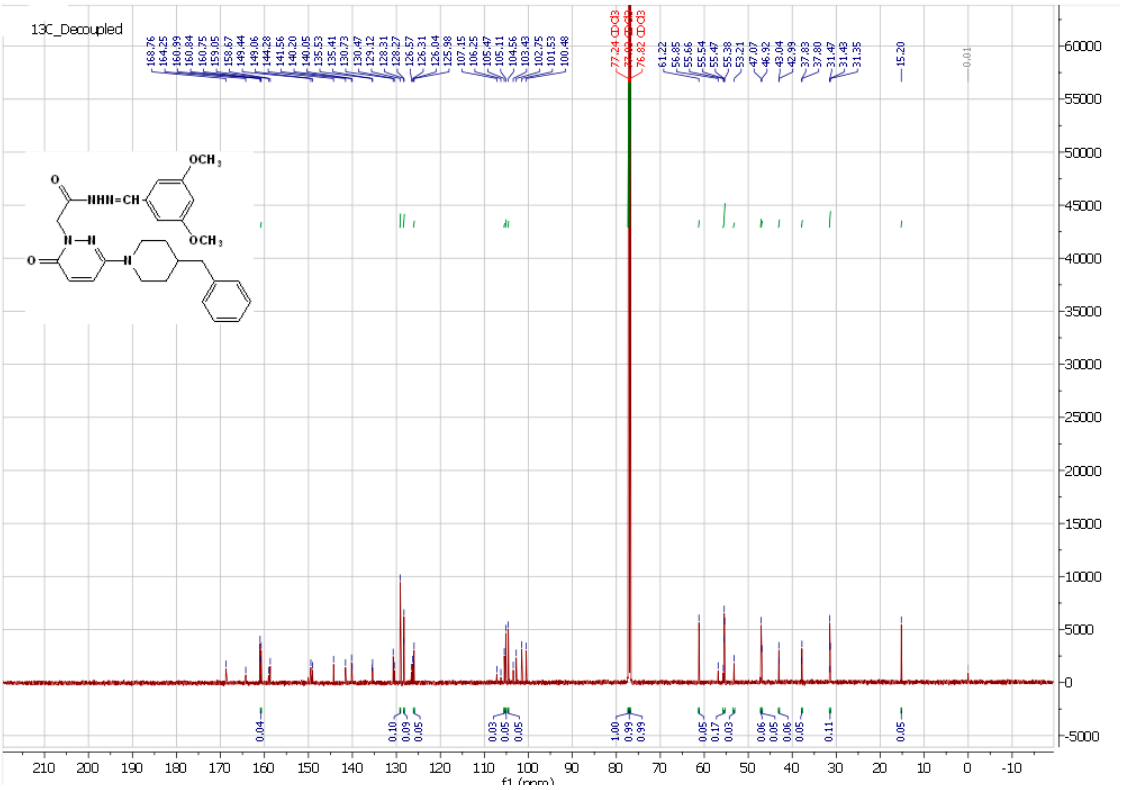

Data 35. HRMS spectra of compound S20

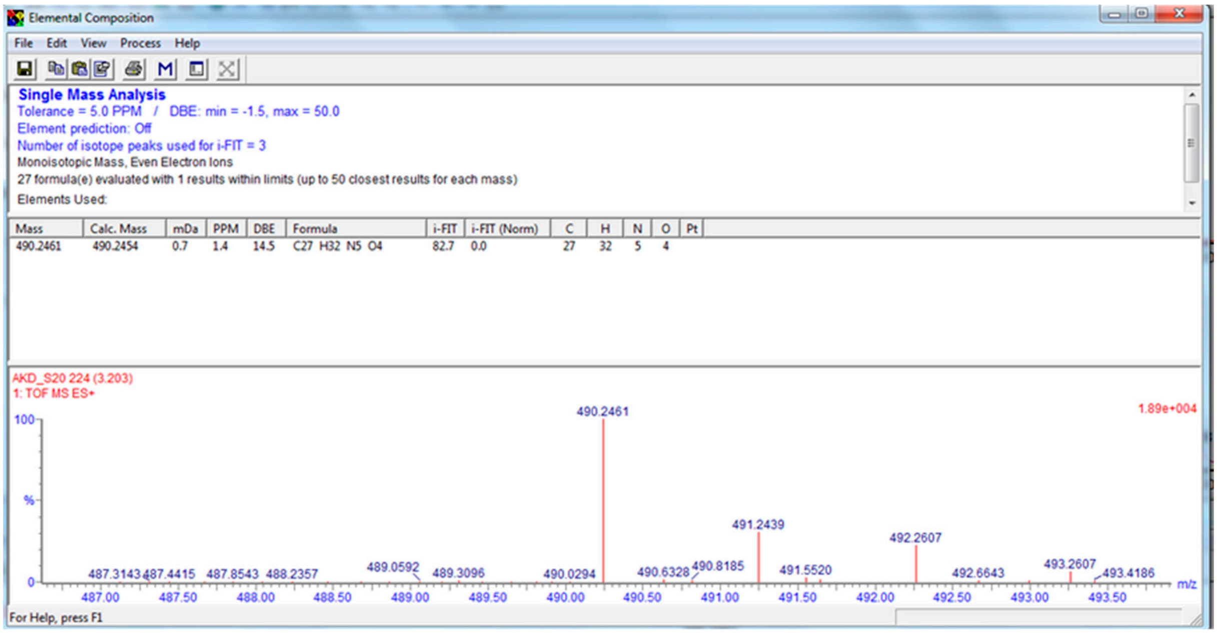

Figure S13. Compound S21

Data 36. ^{1}H-NMR spectra of compound S21

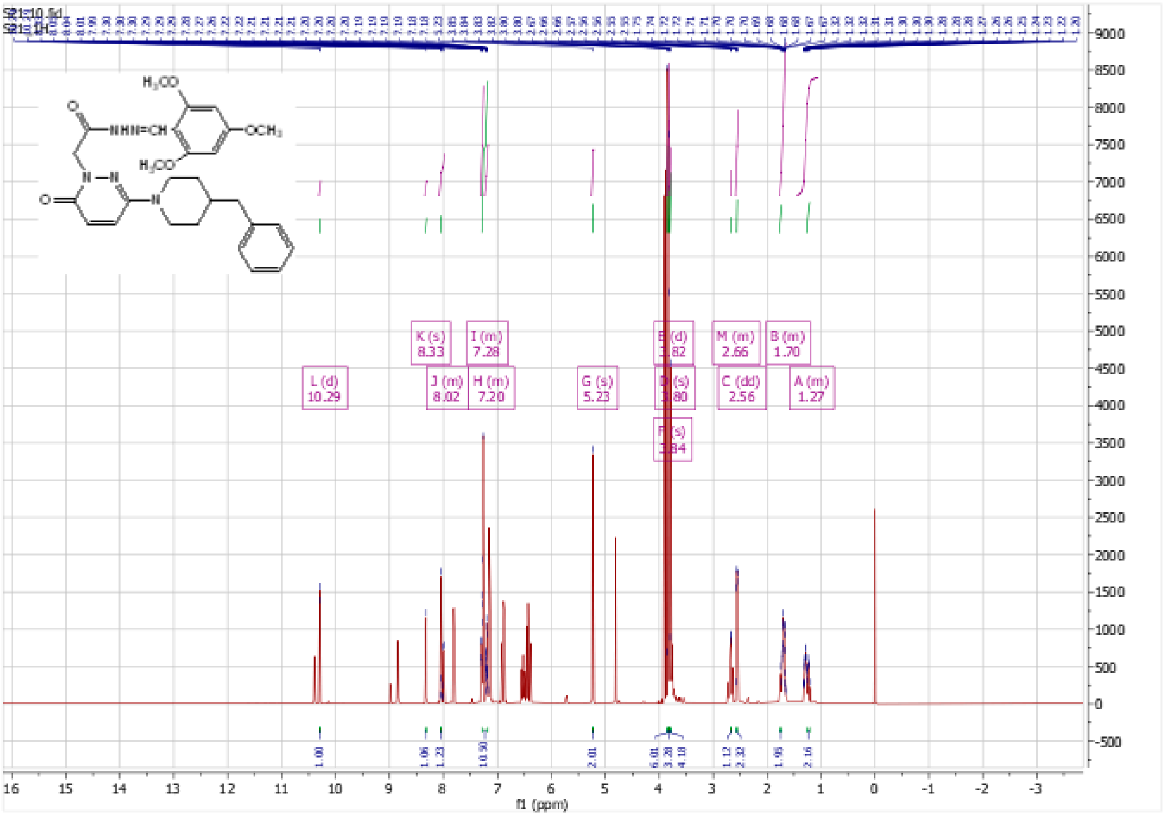

Data 37. ^{13}C-NMR spectra of compound S21

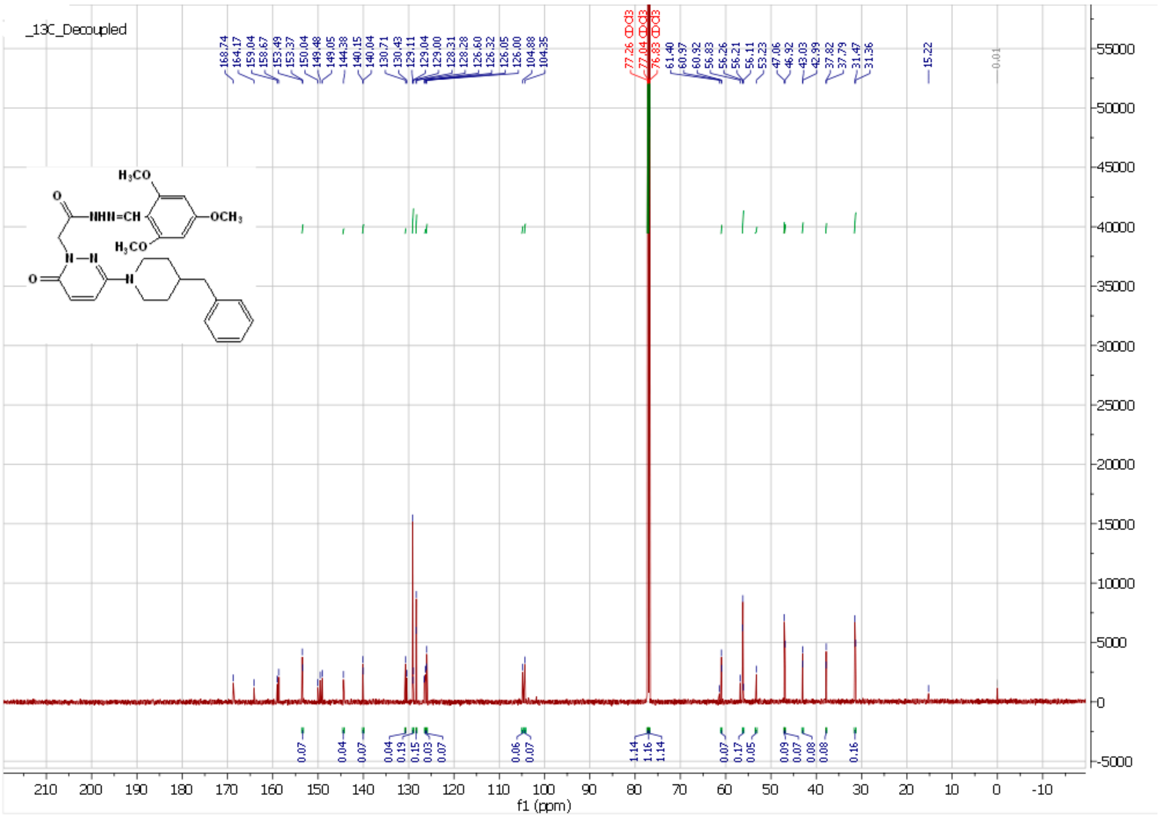

Data 38. HRMS spectra of compound S21

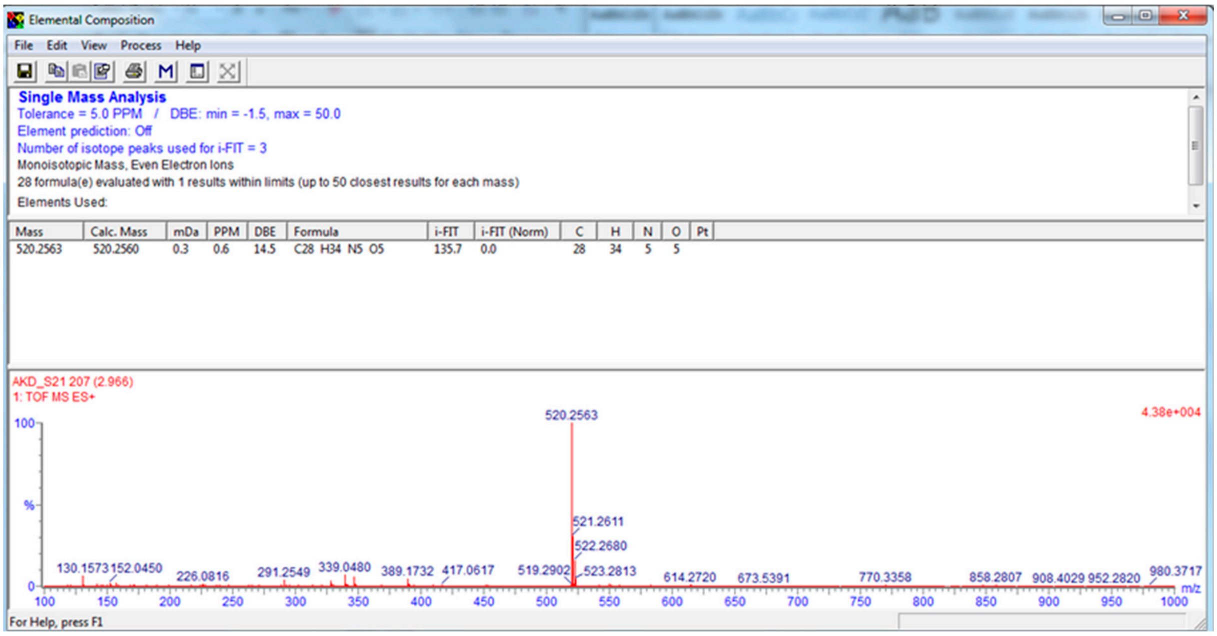

Figure S14. Compound S22

Data 39. ^{1}H-NMR spectra of compound S22

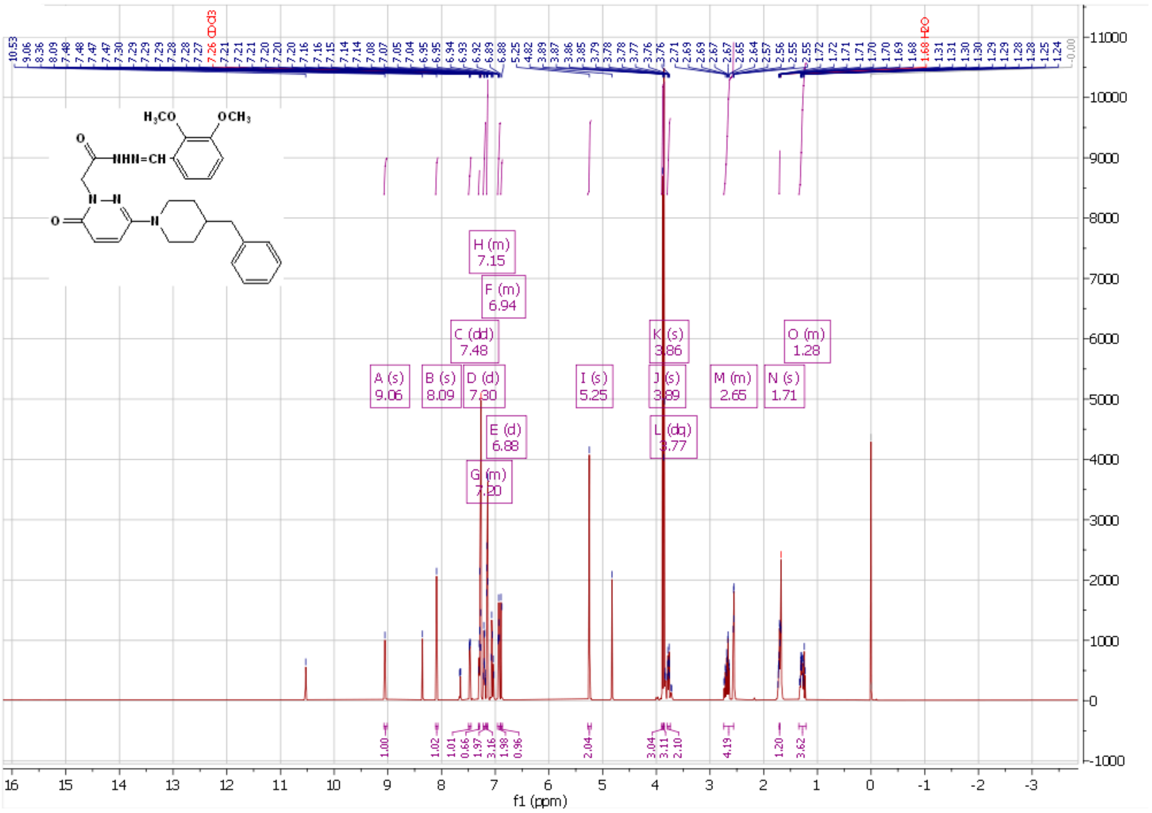

Data 40. ^{13}C-NMR spectra of compound S22

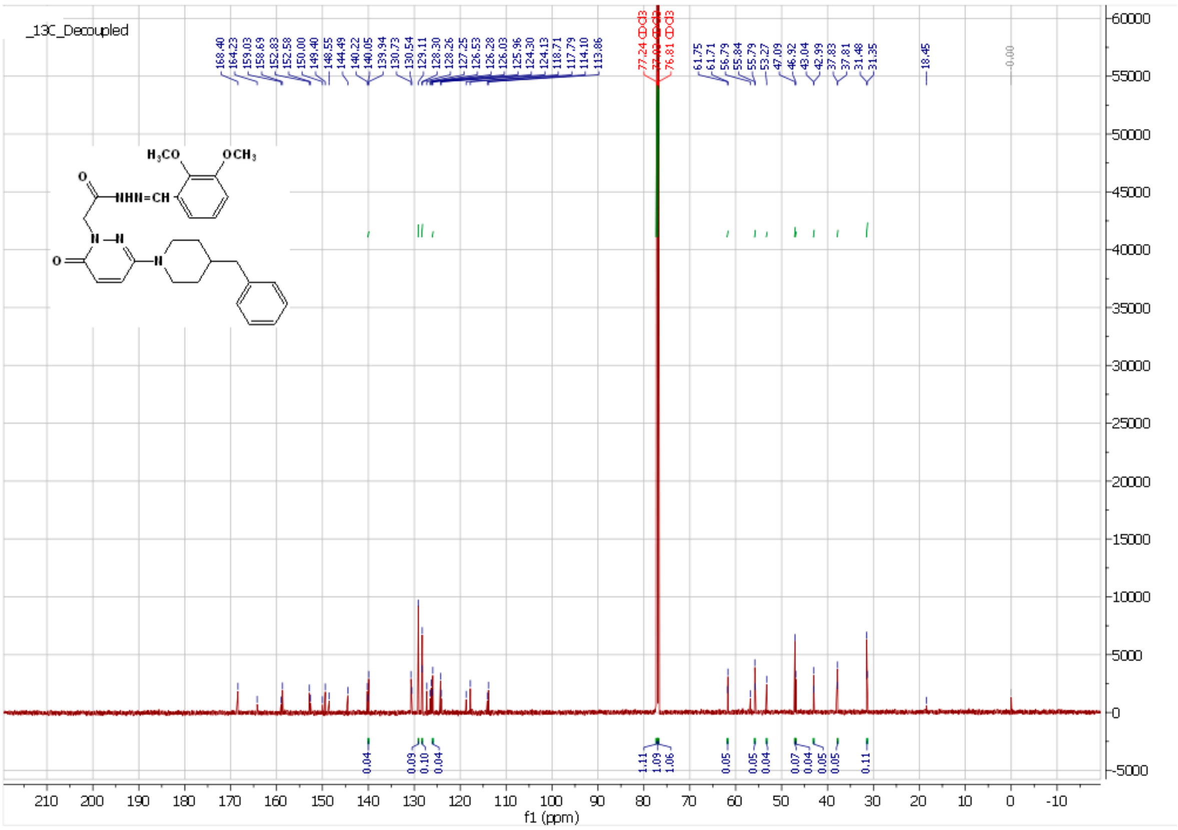

Data 41. HRMS spectra of compound S22

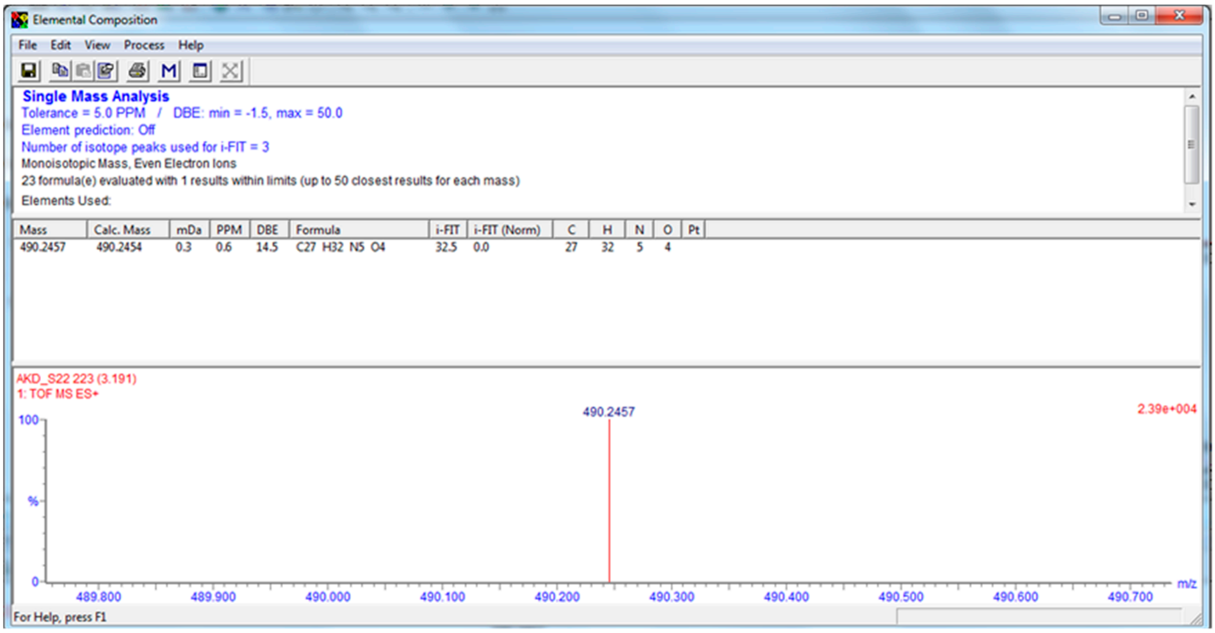

Figure S15. Compound S23

Data 42. ^{1}H-NMR spectra of compound S23

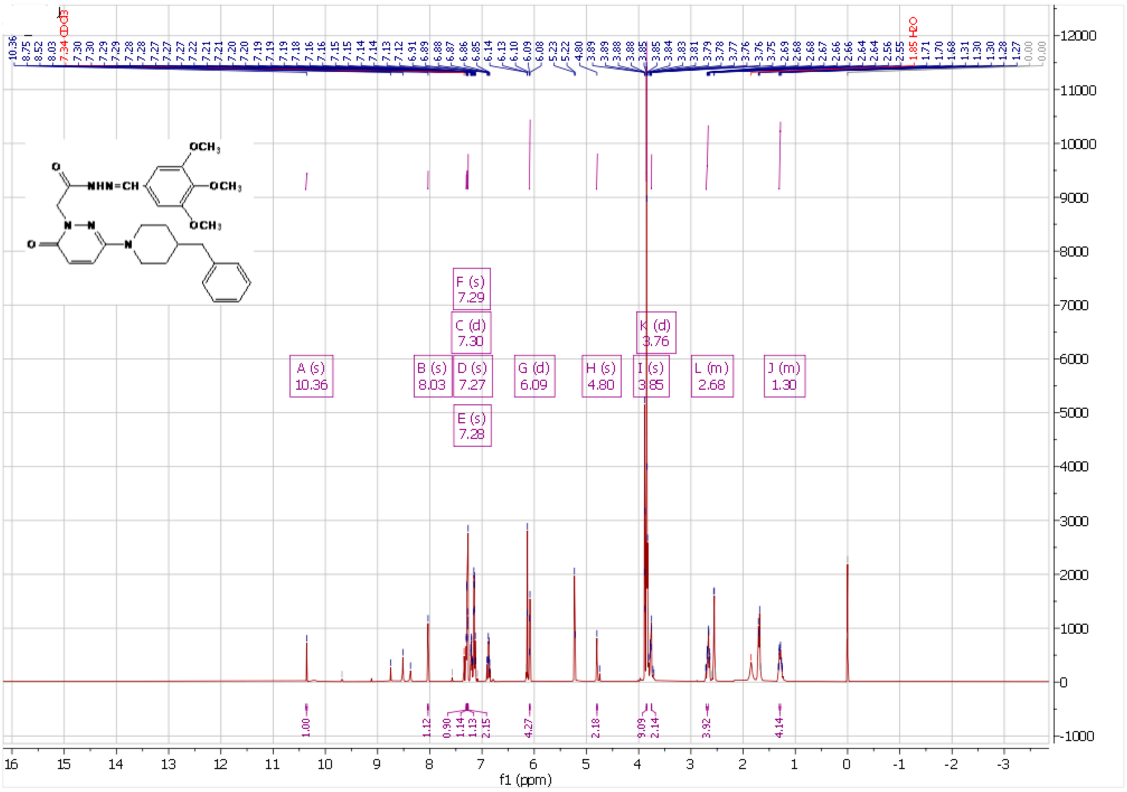

Data 43. ^{13}C-NMR spectra of compound S23

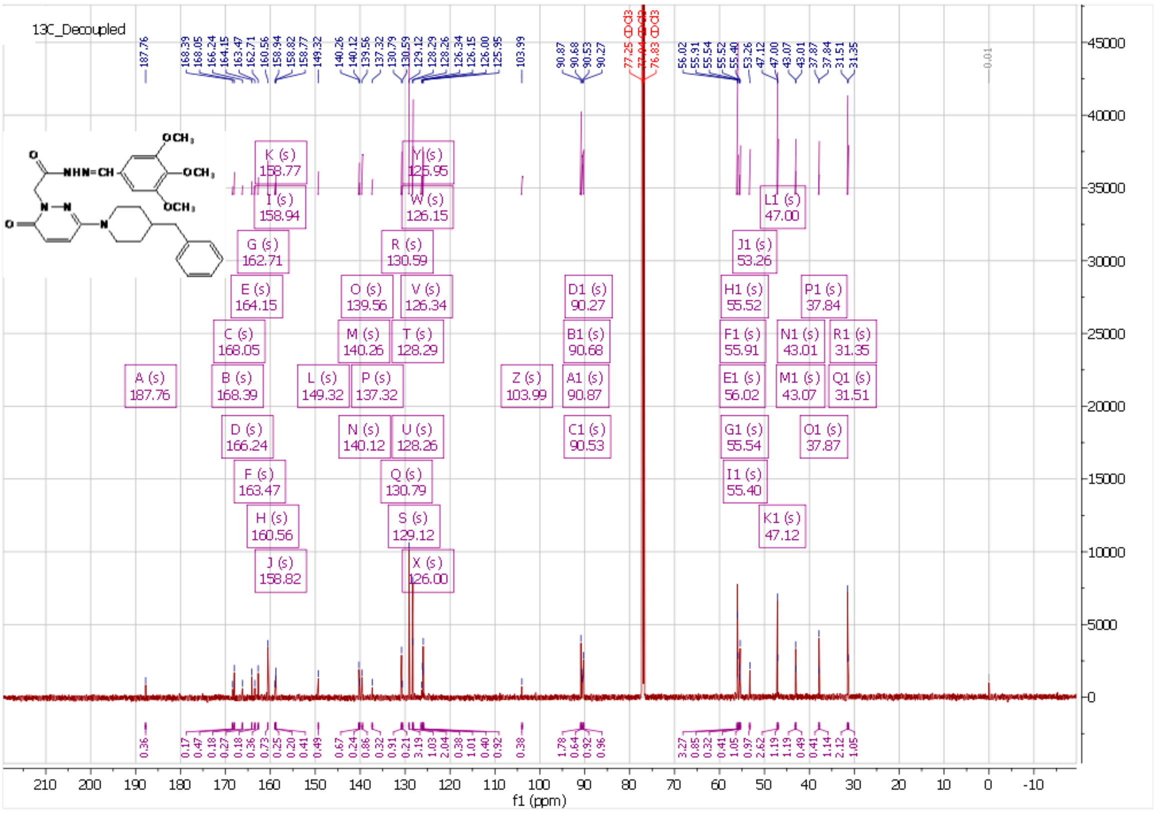

Data 44. HRMS spectra of compound S23

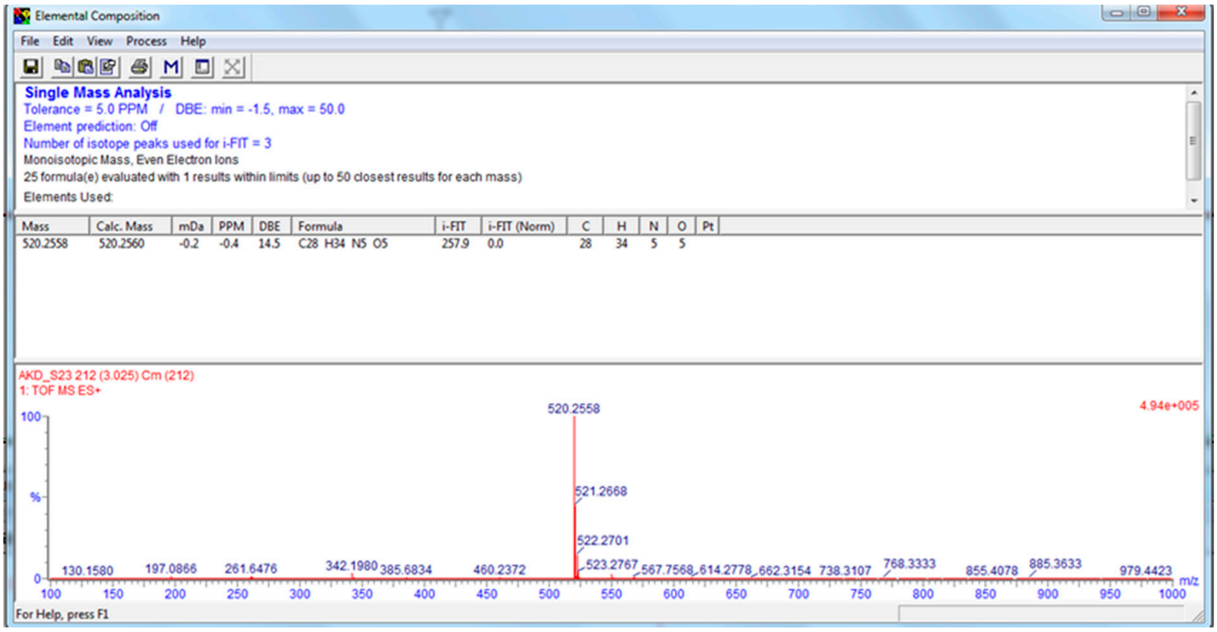

Figure S16. Compound S24

Data 45. ^{1}H-NMR spectra of compound S24

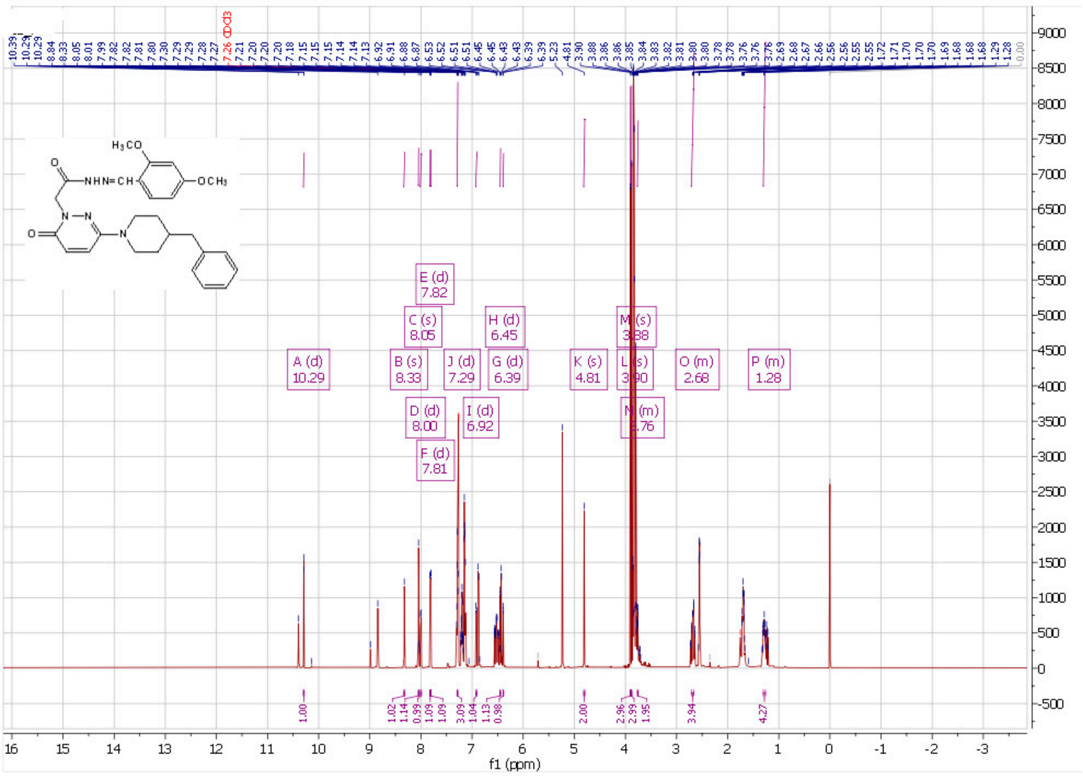

Data 46. ^{13}C-NMR spectra of compound S24

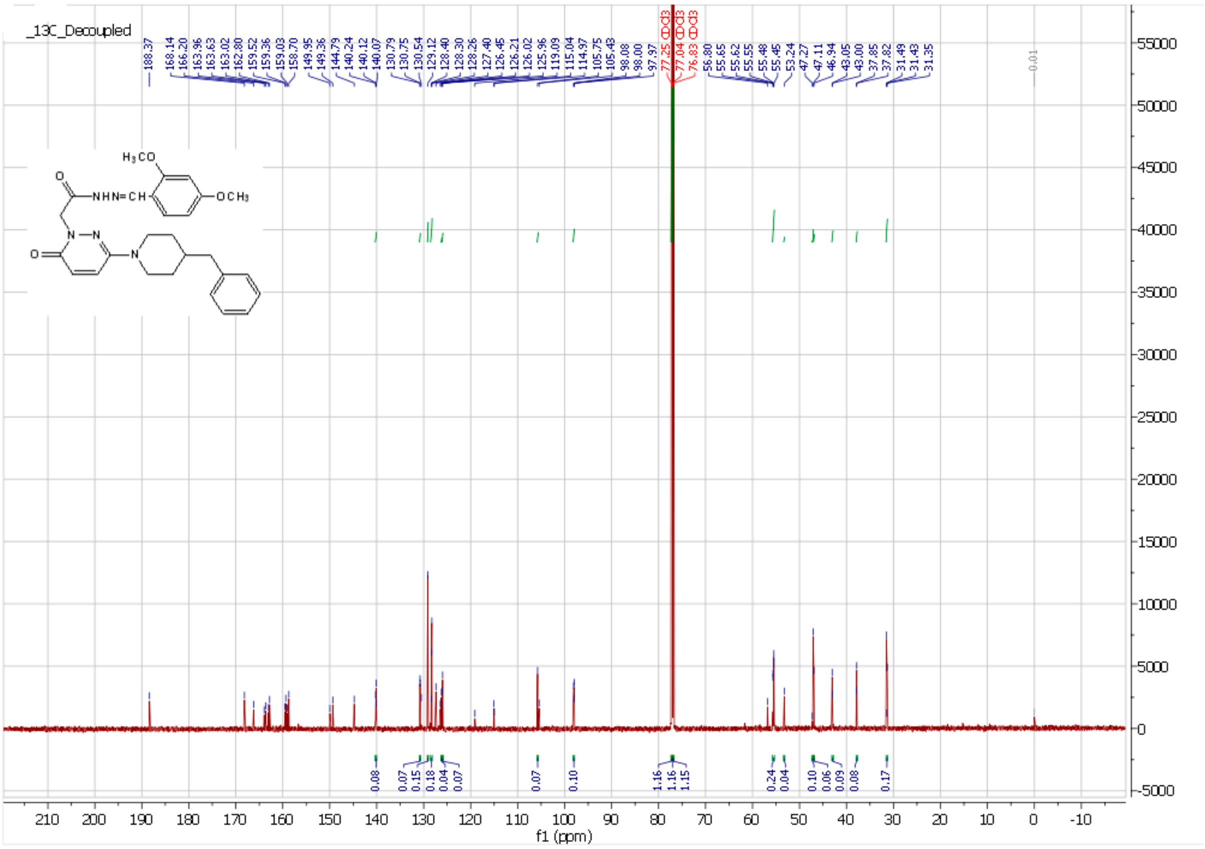

Data 47. HRMS spectra of compound S24

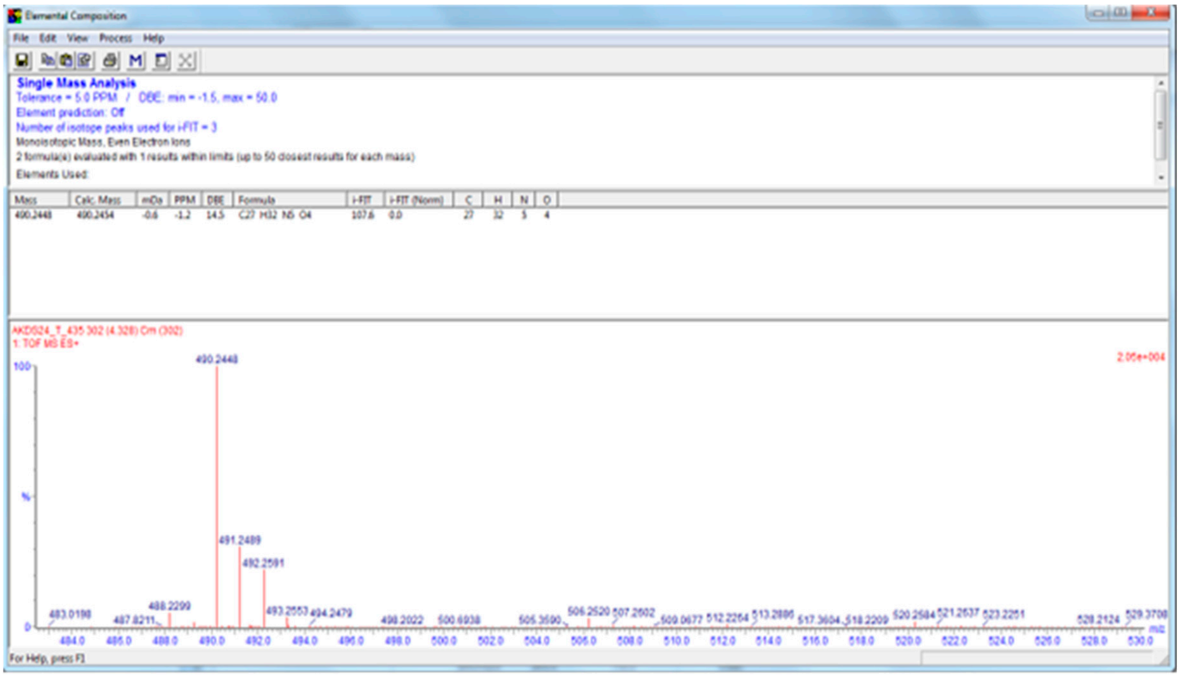

Supplement: Supplementary file 1 [file molecules-29-03097-s001.zip › molecules-3076801-supplementary.pdf]
